# Assessing the role of blood pressure in amyotrophic lateral sclerosis: a Mendelian randomization study

**DOI:** 10.1186/s13023-022-02212-0

**Published:** 2022-02-16

**Authors:** Kailin Xia, Linjing Zhang, Lu Tang, Tao Huang, Dongsheng Fan

**Affiliations:** 1 Department of Neurology, Peking University Third Hospital, Garden North Road No. 49, Beijing, 100191 China; 2 Beijing Municipal Key Laboratory of Biomarker and Translational Research in Neurodegenerative Diseases, Beijing, China; 3 Key Laboratory for Neuroscience, National Health Commission/Ministry of Education, Peking University, Beijing, China; 4 Department of Epidemiology and Biostatistics, School of Public Health, Peking University, Beijing, China; 5 Key Laboratory of Molecular Cardiovascular Sciences (Peking University), Ministry of Education, Beijing, China

**Keywords:** Amyotrophic lateral sclerosis, Blood pressure, Calcium channel blocker, Mendelian randomization

## Abstract

**Background:**

Observational studies have suggested a close but controversial relationship between blood pressure (BP) and amyotrophic lateral sclerosis (ALS). It remains unclear whether this association is causal. The authors employed a bidirectional two-sample Mendelian randomization (MR) approach to evaluate the causal relationship between BP and ALS. Genetic proxies for systolic blood pressure (SBP), diastolic blood pressure (DBP), antihypertensive drugs (AHDs), ALS, and their corresponding genome-wide association study (GWAS) summary datasets were obtained from the most recent studies with the largest sample sizes. The inverse variance weighted (IVW) method was adopted as the main approach to examine the effect of BP on ALS and four other MR methods were used for sensitivity analyses. To exclude the interference between SBP and DBP, a multivariable MR approach was used.

**Results:**

We found that genetically determined increased DBP was a protective factor for ALS (OR = 0.978, 95% CI 0.960–0.996, *P* = 0.017) and that increased SBP was an independent risk factor for ALS (OR = 1.014, 95% CI 1.003–1.025, *P* = 0.015), which is supported by sensitivity analyses. The use of calcium channel blocker (CCB) showed a causal relationship with ALS (OR = 0.985, 95% CI 0.971–1.000, *P* = 0.049). No evidence was revealed that ALS caused changes in BP.

**Conclusions:**

This study provides genetic support for a causal effect of BP and ALS that increased DBP has a protective effect on ALS, and increased SBP is a risk factor for ALS, which may be related to sympathetic excitability. Blood pressure management is essential in ALS, and CCB may be a promising candidate.

**Supplementary Information:**

The online version contains supplementary material available at 10.1186/s13023-022-02212-0.

## Background

Amyotrophic lateral sclerosis (ALS) is a rare fatal neurodegenerative disorder with particularly severe loss of motor neurons and consequent muscle weakness and wasting [1]. The median survival of ALS patients is three years after symptom onset, and death is mainly due to respiratory failure [2]. The poor prognosis of ALS has placed a massive burden on society and the economy; however, the cause and pathogenesis of ALS remain largely elusive. Approximately 10% of ALS cases have a positive family history (fALS), whereas others are sporadic ALS (sALS). Therefore, there is a great need to identify causal risk factors for ALS and corresponding strategies in disease prevention. Recently, an increasing number of nonmotor symptoms have been reported in ALS, which have mainly been neglected by doctors and recalled by more than 60% of patients [3, 4], providing new directions for further research.

Hypertension is one of the critical risk factors for cardiovascular events and chronic kidney disease, contributing to the global disease burden most significantly [5]. Hypertension is largely determined by inheritance with a heritability of approximately 50% [6]. Epidemiological studies revealed abnormal blood pressure values in ALS patients compared with healthy controls [7, 8]. However, the effect of blood pressure on ALS is controversial. A study reported hypertension as a risk factor for ALS due to the association between the long duration of hypertension and poor ALS survival in univariate analysis [9]. Some studies suggested that hypertension was associated with a delay in the age of ALS onset and identified hypertension as a protective factor for ALS risk [10, 11]. Due to these inconsistent observational studies being susceptible to the influence of confounders, selection biases, and reverse causality, the true association between blood pressure and ALS remains largely ambiguous. If increased blood pressure is a causal risk factor for ALS, it would help to better understand the pathophysiology of ALS. Similarly, interventions targeting hypertension could be a promising option for ALS prevention.

Mendelian randomization (MR) is a novel statistical approach for assessing the causal relationship between an exposure and an outcome depending on genetic variances as instrumental variables (IVs) [12]. By simulating randomized controlled trials (RCTs) with naturally grouped risk alleles, the MR approach can produce weighted controls to account for reverse causality and confounders [13]. Therefore, in the present study, we employed a bidirectional two-sample MR study with the most recent genome-wide association studies (GWASs) summary-level data to systematically decipher the causal association between blood pressure and ALS and elucidate the influences of antihypertensive drugs on ALS risk.

## Methods and materials

### Data sources

#### GWAS summary data collection

To assess blood pressure comprehensively and enhance statistical power, we regarded blood pressure as a continuous variable instead of a binary variable that considered the presence or absence of hypertension [14]. Systolic blood pressure (SBP) and diastolic blood pressure (DBP) were included as separate exposures. We obtained their summary-level data from the largest GWASs involving more than 757,600 European individuals, which are meta-analyses on the data from the UK Biobank and the International Consortium for Blood Pressure after adjusting for age and sex [15]. Blood pressure was measured by automated measurement or manual measurement. The mean SBP was 141.1 mmHg (standard deviation (SD) = 20.7), and the mean DBP was 84.3 mmHg (SD = 11.3).

We attempted to study the influence of antihypertensive drugs on ALS. Proxies for common antihypertensive drugs (including angiotensin-converting enzyme inhibitor (ACEI), angiotensin receptor blocker (ARB), β-blocker (BB), and calcium channel blocker (CCB)) were determined by DrugBank (https://www.drugbank.ca/) and GeneCards (https://www.genecards.org/).

We exploited the most recent ALS GWAS from a large-scale study performed by Nicolas et al*.* with 80,610 European individuals, where the proportion of ALS cases was 0.258 [16]. All the patients had disease onset after age 18 years and were diagnosed at probable or definite levels according to the El Escorial criteria.

#### IV selection

Single-nucleotide polymorphisms (SNPs) independently (r^2^ < 0.001) associated with blood pressure at the genome-wide significance level (*P* < 5E−8) were strictly selected as IVs, and IVs for antihypertensive drugs were all significantly associated with SBP (*P* < 5E−8) and in relatively modest linkage disequilibrium (LD) (r^2^ < 0.4), both as described previously [17], which increased the proportion of phenotypic variance explanation and statistical power. The genetic contributions of each allele change in SBP and DBP were 0.016 and 0.025, respectively.

IVs absent in the ALS dataset were replaced with proxies in strong LD (r^2^ > 0.9) by searching the publicly available online tool SNiPA (http://snipa.helmholtz-muenchen.de/snipa3/). Those without reported proxies were excluded from downstream MR analysis. Due to the requirements of MR approaches, the exposure would be removed when its available IVs were less than two. Altogether, 400 SNPs were identified as IVs for SBP, 397 SNPs were identified as IVs for DBP, 47 SNPs were identified as IVs for CCB, and 5 SNPs were identified as IVs for ALS. An additional table showed this in more detail (see Additional file 1). ACEI, ARB, and BB were removed due to insufficient IVs. In multivariable MR (MVMR) analysis (see below), 62 SNPs for SBP and 30 SNPs for DBP were excluded because they were palindromic with intermediate allele frequencies.

#### Two-sample MR

The theoretical basis of MR research relies on three assumptions: *assumption 1,* the selected genetic variances are not related to other confounders; *assumption 2*, the selected genetic variances are significantly related to exposure; and *assumption 3*, the selected genetic variances are significantly related to the risk of outcome only through the pathway of exposure [18]. The strict selection of IVs satisfied assumption 1. Assumptions 2 and 3 were met through various MR approaches.

**Fig. 1:**
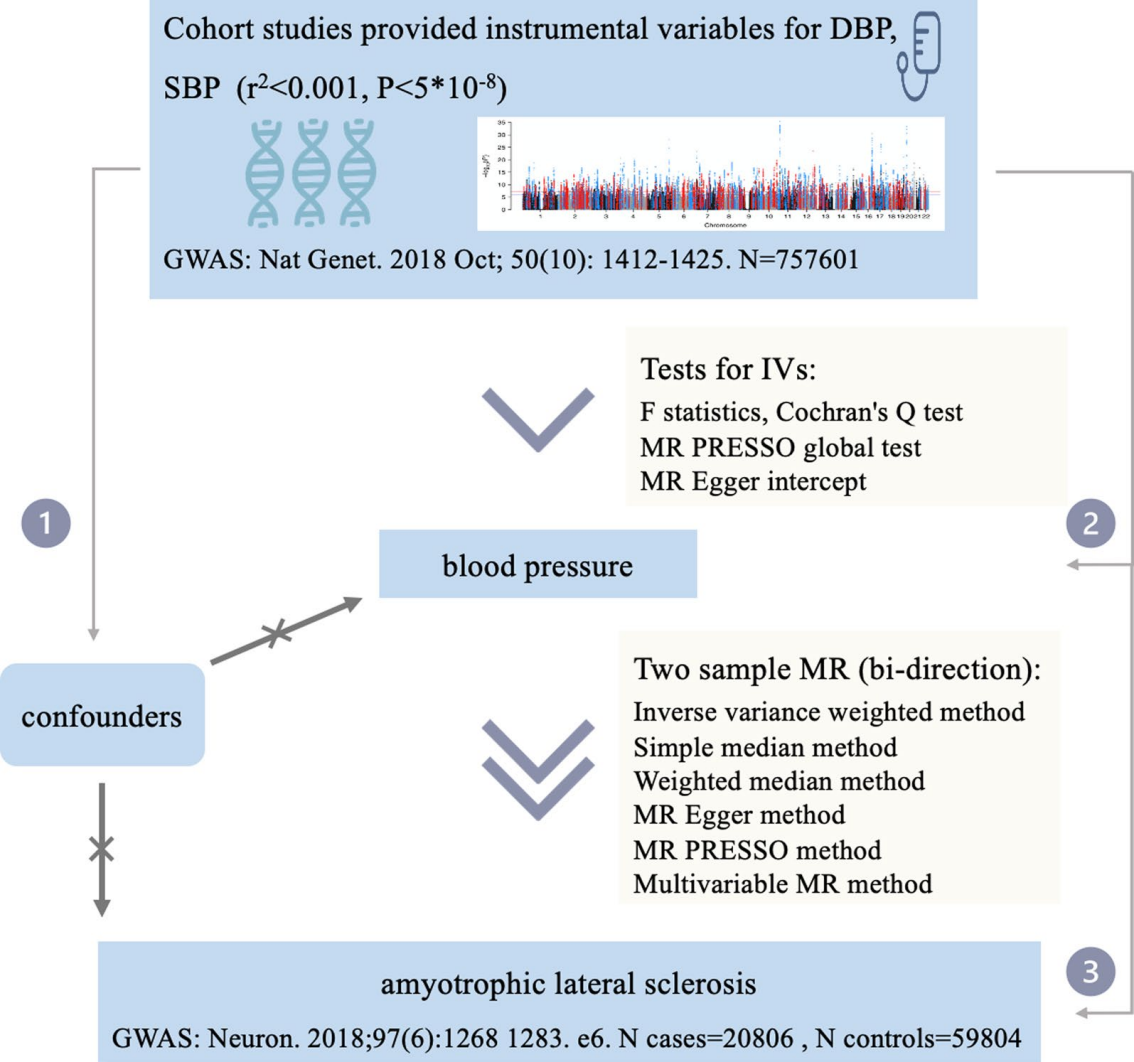
Flow chart showing the process for the Mendelian randomization analyses. The number in the line indicates 3 key assumptions for MR. Assumption 1: The selected genetic variances are not related to other confounders. Assumption 2: The selected genetic variances are significantly related to the exposure. Assumption 3: The selected genetic variances are significantly related to the risk of the outcome only through the pathway from exposure. GWAS, genome-wide association studies; MR, Mendelian randomization; SNP, single-nucleotide polymorphism; DBP, diastolic blood pressure; SBP, systolic blood pressure

We implemented the multiplicative random effects inverse variance weighted (IVW) method as the main approach to examine the overall causal relationship between exposure and ALS based on the effect of IVs on exposure and the effect of IVs on ALS [19]. To validate the results from the IVW method, we applied the weighted median method, simple median method [20], MR Egger method [21] and MR-PRESSO method as sensitivity analyses. To evaluate the potential pleiotropy of IVs, MR Egger regression, which accounts for the presence of pleiotropy when the intercept significantly deviates from the origin, and MR-PRESSO analysis, which was used to detect the influence of outliers [22], were employed. The heterogeneity of SNPs utilized in IVW estimates was tested by Cochran's Q test, which suggests apparent heterogeneity when it is lower than the significant *P* value. Leave-one-out analysis was employed to evaluate the possibility of results being driven by a single SNP. We also calculated F statistics for IVs to demonstrate whether they are strong instruments. Given the close correlation between SBP and DBP, a multivariable MR (MVMR) approach was adopted to diminish the interference between them. Specifically, the causal association between SBP and ALS was estimated regarding DBP as a covariate, and the association between DBP and ALS was estimated with SBP as a covariate. In addition, reverse causation between blood pressure and ALS was assessed by bidirectional MR analysis, which made ALS the exposure and blood pressure the outcome. The process is shown in Fig. 1.

*P* values less than 0.05/3 were considered significant with Bonferroni correction, and a *P* value between 0.017 and 0.05 was regarded as a suggestive significance level. All analyses were carried out in R software version 3.6.3 by the "TwoSampleMR" package (version 0.5.6) [23] and the "MR-PRESSO" package (version 1.0) [22].

## Results

**Fig. 2:**
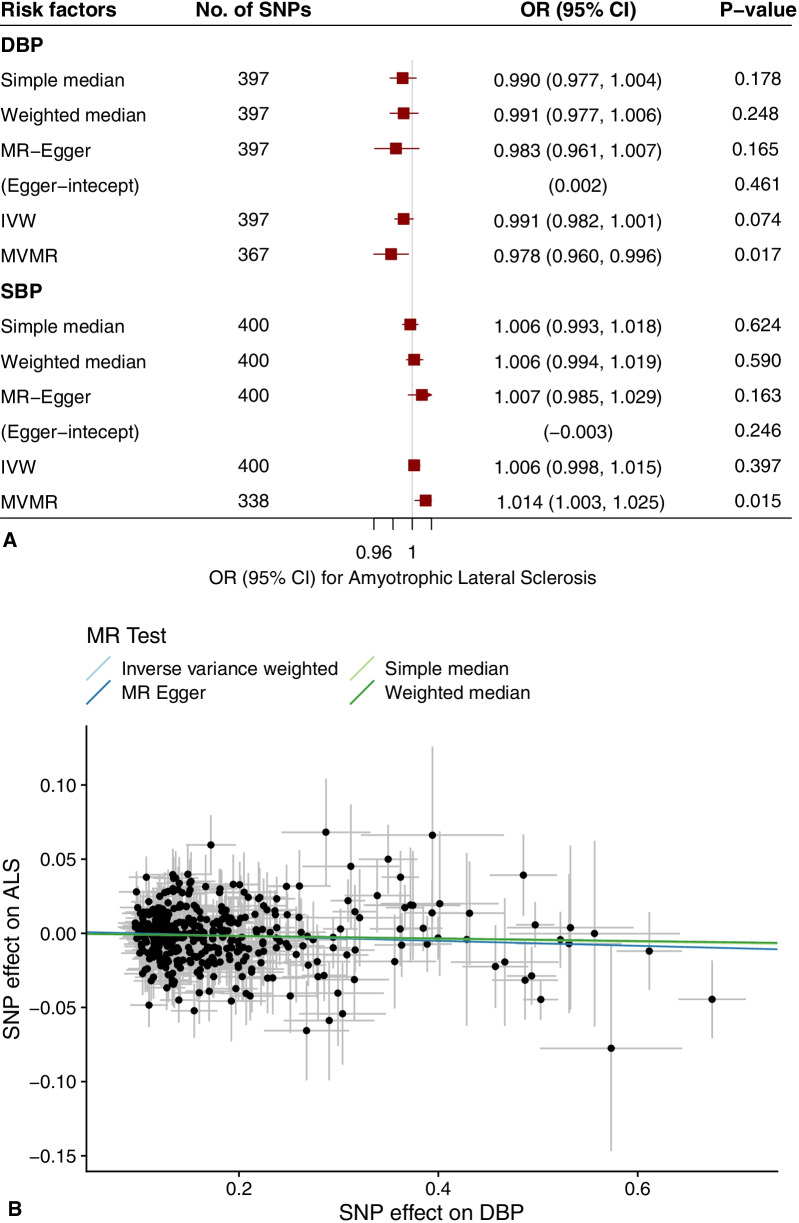

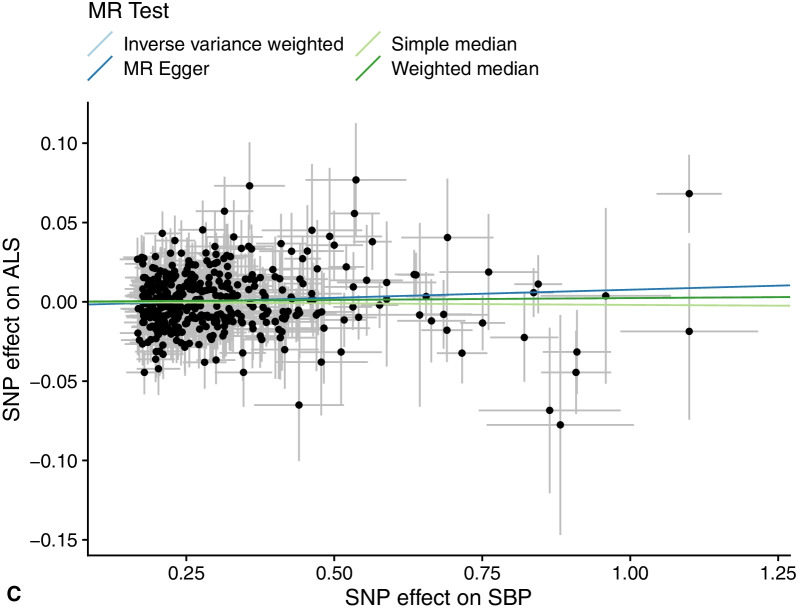
The relationship between blood pressure and ALS. **a** The main results of the effects of 1 genetically predicted SD unit increase in blood pressure on ALS via MR approaches. **b** Scatter plot of the SNP effects on DBP versus ALS, with the slope of each line corresponding to the estimated MR effect per method. **c** Scatter plot of the SNP effects on SBP versus ALS, with the slope of each line corresponding to the estimated MR effect per method. OR, odds ratio; CI, confidence interval; MR, Mendelian randomization; SNP, single-nucleotide polymorphism; DBP, diastolic blood pressure; SBP, systolic blood pressure; ALS, amyotrophic lateral sclerosis

**Fig. 3:**
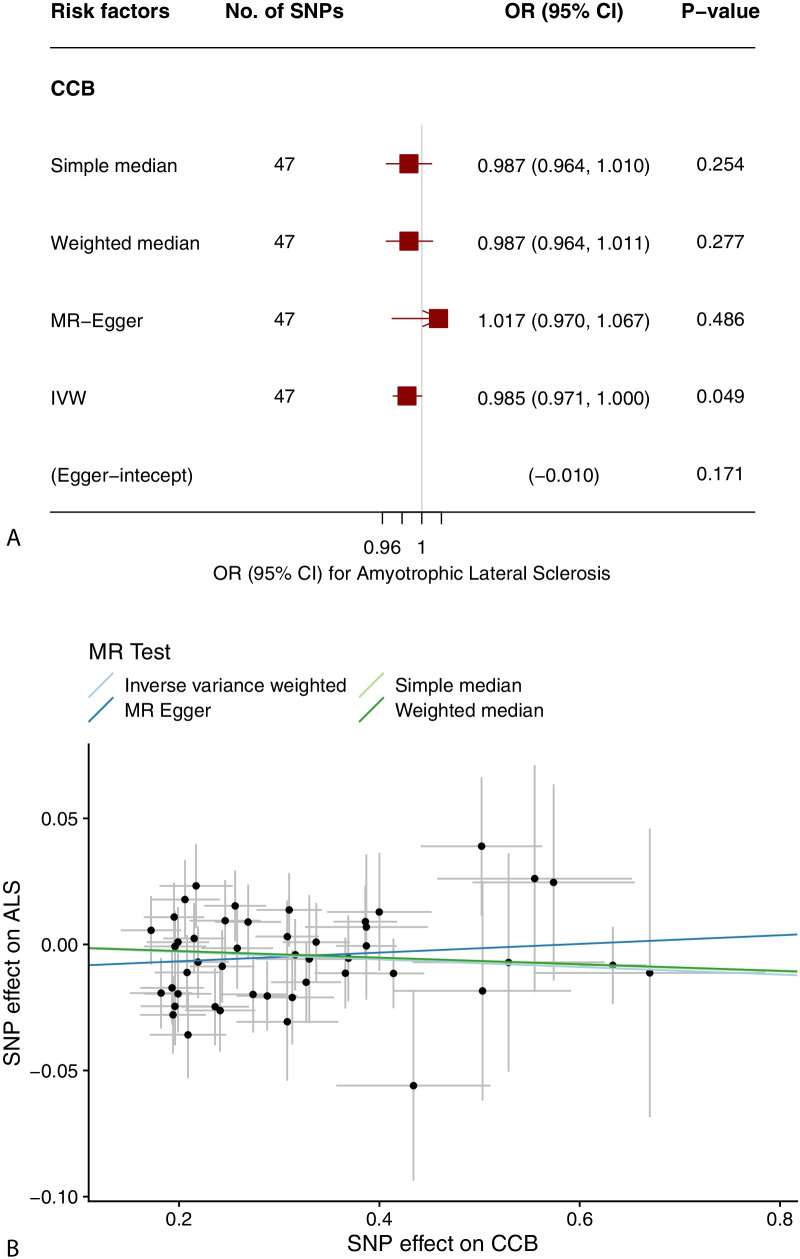
The relationship between CCB and ALS. **a** The main results of the effects of genetically predicted CCB on ALS via MR approaches. **b** Scatter plot of the SNP effects on CCB versus ALS, with the slope of each line corresponding to the estimated MR effect per method. OR, odds ratio; CI, confidence interval; MR, Mendelian randomization; SNP, single-nucleotide polymorphism; CCB, calcium channel blocker; ALS, amyotrophic lateral sclerosis

In our study, proxies related to SBP, DBP, CCB, and ALS were utilized to investigate the relationship between blood pressure and ALS using five MR methods. The detailed results are displayed in Tables 1 and 2, and the main results are visualized in Figs. 2 and 3.

**Table 1:** Summary of the causal effects of each trait on ALS via different MR methods

	DBP	SBP	CCB
N SNPs	397	400	47
F statistics	31,000	29,400	3180
Cochran’s Q			
Q	446.290	431.440	40.603
*p* value	0.041	0.127	0.697
Simple median			
OR (95% CI)	0.990 (0.977, 1.004)	1.006 (0.993, 1.018)	0.987 (0.964, 1.010)
*P *value	0.178	0.624	0.254
Weighted median			
OR (95% CI)	0.991 (0.977, 1.006)	1.006 (0.994, 1.019)	0.987 (0.964, 1.011)
*P* value	0.248	0.590	0.277
MR Egger			
OR (95% CI)	0.983 (0.961, 1.007)	1.007 (0.985, 1.029)	1.017 (0.970, 1.067)
*P* value	0.165	0.163	0.486
Inverse variance weighted-mre			
OR (95% CI)	0.991 (0.982, 1.001)	1.006 (0.998, 1.015)	0.985 (0.971, 1.000)
*P* value	0.074	0.397	0.049
MR Egger			
Intercept	0.002	− 0.003	− 0.010
*P* value	0.461	0.246	0.171
MR-PRESSO			
Outlier-corrected	NA	NA	NA
Global test *P* value	0.042	0.140	0.720
Distortion test	NA	NA	NA
Multivariable MR			
OR (95% CI)	0.978 (0.960, 0.996)	1.014 (1.003, 1.025)	NA
*P* value	0.017	0.015	NA

*DBP* diastolic blood pressure, *SBP* systolic blood pressure, *ALS* amyotrophic lateral sclerosis, *CCB* calcium channel blocker, *OR* odds ratio, *CI* confidence interval, *MR* Mendelian randomization, *SNP* single-nucleotide polymorphism

**Table 2:** Summary of the causal effects of ALS on blood pressure via MR methods

	DBP	SBP
N SNPs	5	5
Cochran’s Q		
Q	7.525	7.002
*p* value	0.111	0.136
Simple median		
OR (95% CI)	1.069 (0.877, 1.302)	1.22 (0.863, 1.726)
*P* value	0.511	0.260
Weighted median		
OR (95% CI)	1.094 (0.922, 1.297)	1.265 (0.934, 1.713)
*P* value	0.304	0.129
MR Egger		
OR (95% CI)	1.349 (0.863, 2.108)	1.686 (0.755, 3.765)
*P* value	0.280	0.292
Inverse variance weighted-mre		
OR (95% CI)	1.024 (0.841, 1.246)	1.126 (0.809, 1.566)
*P* value	0.815	0.482
MR Egger		
Intercept	− 0.045	− 0.066
*P* value	0.278	0.360
MR-PRESSO		
Outlier-corrected	NA	NA
Global test *P* value	0.203	0.223
Distortion test	NA	NA

*DBP* diastolic blood pressure, *SBP* systolic blood pressure, *ALS* amyotrophic lateral sclerosis, *CCB* calcium channel blocker, *OR* odds ratio, *CI* confidence interval, *MR* Mendelian randomization, *SNP* single-nucleotide polymorphism

We found that increased DBP had a potential protective effect on ALS (IVW-odds ratio (OR) = 0.991, 95% confidence interval (CI) 0.982–1.001, *P* = 0.074) (Table 1, Fig. 2). The simple median method (OR = 0.990, 95% CI 0.977–1.004, *P* = 0.178) and the MR Egger method (OR = 0.983, 95% CI 0.961–1.007, *P* = 0.165) generated similar results with wider CIs and less precision. No influence of outliers was detected. The MR PRESSO global test provided suggestive horizontal pleiotropy for IVs (*P* = 0.042), which was not supported by the MR Egger intercept (intercept = 0.002, *P* = 0.461). The heterogeneity of IVs was observed by Cochran's Q test (Q = 446.290, *P* = 0.041). When we adjusted SBP, the effect of DBP on ALS was enhanced. The risk of ALS was alleviated by 2.2% (OR = 0.978, 95% CI 0.960–0.996, *P* = 0.017) with a per genetically predicted SD increase in DBP (approximately 11 mmHg). In the bidirectional causal relationship exploration, no evidence was found that ALS caused changed DBP values (IVW-OR = 1.024, 95% CI 0.841–1.246, *P* = 0.815) (Table 2).

With an overview analysis of the association between SBP and ALS, SBP was suggested to have a null genetic relationship with ALS (IVW-OR = 1.006; 95% CI 0.998–1.015; *P* = 0.397) (Table 1, Fig. 2). Sensitivity analyses generated analogical results. We observed no obvious horizontal pleiotropy of the IVs with the MR Egger method (intercept = − 0.003, *P* = 0.246). No instrument outliers existed, and Cochran's Q test detected no heterogeneity. After DBP was adjusted, we observed a significant risk effect of SBP on ALS, which elevated ALS risk by 1.4% per genetically predicted unit increase (OR = 1.014, 95% CI 1.003–1.025 *P* = 0.015). No modifying role of ALS on SBP values was manifested (OR, 1.126, 95% CI 0.809–1.567, *P* = 0.482) (Table 2).

Considering the deleterious effect of high SBP on ALS, we probed the role of antihypertensive drugs on ALS. Owing to the limited numbers of IVs, ACEI, ARB, and BB were excluded from the downstream analyses. The IVW approach indicated a suggestive protective role of CCB on ALS (OR = 0.985; 95% CI 0.971–1.000; *P* = 0.049), which was not verified by additional MR analyses. No influence of the outliers, heterogeneity, or horizontal pleiotropy were detected (Table 1, Fig. 3).

## Discussion

Based on large-scale blood pressure GWASs and ALS GWAS, we conducted a two-sample bidirectional MR study and found that an increased DBP may be a protective factor for ALS, while SBP may be a risk factor for ALS in the European population, shedding light on the importance of nonmotor systems in ALS pathogenesis. Furthermore, CCB was suggested to have a protective effect on ALS in our study, which may modify ALS management.

An observational study and animal study noted a lower blood pressure in ALS patients than in healthy controls [8, 24]. However, the effect of abnormal blood pressure on ALS remains inconsistent. We utilized the MR framework to elucidate the causal relationship between blood pressure and ALS. With the IVW approach, DBP was assessed to play a protective role on ALS risk with an OR equal to 0.978 after adjusting for SBP, and sensitivity analyses generated results in the same direction. However, there was heterogeneity in the IVs employed for DBP, which may increase the probability of type one error. When investigating the role of SBP in ALS while eliminating the influence of DBP, SBP was identified as a genetically described risk factor for ALS risk. Additional MR analyses verified the robustness of this result. Our results are not fully supported by those in a previous study [25], and we carefully recognized the causal relationship between blood pressure and ALS by doubling the sample size. Furthermore, previous evidence for the relationship between blood pressure and ALS was mainly derived from observational studies, which could not distinguish between abnormal blood pressure causing ALS or ALS status causally influencing blood pressure. To clarify this, we performed a reverse direction MR analysis to determine the effect of ALS on blood pressure values. No results supported the view that ALS can influence blood pressure values. In addition, SBP is the current primary therapeutic target in blood pressure management, and its elevation is more common and critical for cardiovascular outcomes than DBP [26, 27]; therefore, the risk effect of SBP on ALS has received closer attention. We explored the effect of antihypertensive drugs on ALS, hoping to find a promising pharmacological intervention for ALS. It was shown that CCB played a protective role in ALS. The protective effect of ACEI on ALS was also reported in a prior study [28], but we failed to verify this finding due to the limited number of IVs to mimic ACEI. Further studies to confirm these results are of great interest. The ‘gold standard’ for empirically alleviating the concerns of residual confounding and reverse causation in clinical research is an RCT [29]. However, RCTs testing the association between blood pressure and ALS risk have not been implemented, mainly due to ethical issues. Therefore, observational studies with actual patients should be involved in future replication. Because the ALS presymptomatic stage could persist for years [30], cohort studies may provide more credible evidence with a diminished interference of reverse causation than other observational studies [29]. A large-scale population-based prospective cohort can be established, where information about blood pressure values and the use of antihypertensive drugs is collected at baseline. After long-term follow-up, the relationship between the observed phenotypes and ALS can be revealed using various association analyses. Combining these data and the results generated with the MR framework may deliver convincing conclusions in the future.

The potential mechanisms underlying the relationship between blood pressure and ALS remain unclear. The results generated with high-resolution magnetic resonance imaging suggested a link between the combination of low DBP and high SBP and cerebral hypoperfusion [31]. Hypoperfusion can lead to insufficient energy in neurons and trigger neuroinflammation and blood–brain barrier disruption, accelerating the neurodegenerative pathological changes in ALS [32, 33]. In addition, the combination of low DBP and high SBP is associated with increased large arterial stiffness, which attenuates resting cerebral blood flow and the clearance of aggregated proteins [34]. Some sympathetic dysfunctions in ALS have been reported recently, including decreased norepinephrine levels [35], decreased heart rate variability, and degeneration of the cardiac sympathetic nerve [36, 37]. Abnormal sympathetic excitability may also play an essential role in the causal pathway from SBP to ALS, which elevates SBP and aggravates oxygen tension in ALS. Low oxygen tension can cause reductive bond cleavage and an increase in disordered superoxide dismutase 1 protein (SOD1) in ALS patient-derived cells, facilitating disease progression [38].

Interventions relevant to calcium ions (Ca^2+^) seem to be a promising target for ALS management. Nevertheless, caution should be taken when explaining the MR results into expected pharmacologic efficacy attributed to the difference between genetically predicted lifelong exposure to a biomarker and short-term intervention. Ca^2+^ is an essential mediator of cell communication and signal transduction, and calcium channels are widely expressed. Basal intracellular calcium levels were elevated in motor neurons with mutant transactive response DNA-binding protein 43 (TDP43), suggesting altered Ca^2+^ homeostasis in ALS [39]. Aberrant Ca^2+^ levels cause functional defects in lysosomes and autophagic flux, hinder the removal of misfolded toxic proteins, and result in further disease development [40, 41]. The protective effect of CCB on ALS may be directly due to the clearance of accumulated Ca^2+^ from neurons.

Our study is the first to shed light on the interventive effect of blood pressure management in ALS. All the analyses are performed with the largest European-based GWASs. Reliable data sources and study design provide sufficient statistical power. Nevertheless, there are still some shortcomings in this study. The limitations of the methodology have been reviewed elsewhere [42]. Some SNPs overlapped in the IVs for SBP and DBP, which may affect the results; therefore, the MVMR approach, sensitivity analyses, and horizontal pleiotropy tests were adopted to assess the true relationship and detect the robustness of estimates. In addition, both sALS and fALS were involved in the ALS GWAS we employed, aiming to validate previous ALS-causing genes and provide more broadly acceptable conclusions. Although sALS and fALS share similar neuropathological signatures and risk factors, monogenetic causes are associated with specific clinical features. Therefore, targeted MR studies that distinguish fALS and sALS would yield more detailed and tailored results. Because sALS was the overwhelming majority, the previous results we found would not change significantly after removing fALS. For analyses focused on fALS, the newly generated results may be in the same direction as those we reported but with wider CIs due to the limited number of cases. However, these hypotheses cannot be confirmed owing to the lack of available ALS GWAS individual-level data. Moreover, the potential effect of blood pressure on the prognosis of ALS was speculated without MR evidence, similarly due to the lack of relevant GWAS data (e.g., clinical progression pattern, cognitive impairment, and survival).

## Conclusion

This study provides genetic support for a causal effect of BP and ALS that increased DBP has a protective effect on ALS, and increased SBP is a risk factor for ALS, which may be related to sympathetic excitability. Blood pressure management is essential in ALS, and CCB may be a promising candidate.

## Supplementary Information

**Additional file 1: Supplementary Table 1.** Information of Instrumental Variables.

## Data Availability

All data generated or analyzed during this study are included in this published article and its supplementary information files. Codes generated or used during the study are available from the corresponding author by request.

## Abbreviations

ACEI: Angiotensin-converting enzyme inhibitor
AHD: Antihypertensive drug
ALS: Amyotrophic lateral sclerosis
ARB: Angiotensin receptor blocker
BB: β-Blocker
BP: Blood pressure
Ca^2^^+^: Calcium ion
CCB: Calcium channel blocker
CI: Confidence interval
DBP: Diastolic blood pressure
GWAS: Genome-wide association study
IV: Instrumental variable
IVW: Inverse variance weighted method
LD: Linkage disequilibrium
MR: Mendelian randomization
MVMR: Multivariable Mendelian randomization
OR: Odds ratio
RCT: Randomized controlled trial
SBP: Systolic blood pressure
SD: Standard deviation
SNP: Single-nucleotide polymorphism

## References

[1] Pandya VA, Patani R (2019). Decoding the relationship between ageing and amyotrophic lateral sclerosis: a cellular perspective. Brain.

[2] Masrori P, Van Damme P (2020). Amyotrophic lateral sclerosis: a clinical review. Eur J Neurol.

[3] Chiò A, Moglia C, Canosa A, Manera U, Vasta R, Brunetti M (2019). Cognitive impairment across ALS clinical stages in a population-based cohort. Neurology.

[4] Chiò A, Mora G, Lauria G (2017). Pain in amyotrophic lateral sclerosis. Lancet Neurol.

[5] GBD 2017 Risk Factor Collaborators (2018). Global, regional, and national comparative risk assessment of 84 behavioural, environmental and occupational, and metabolic risks or clusters of risks for 195 countries and territories, 1990–2017: a systematic analysis for the Global Burden of Disease Study 2017. Lancet.

[6] Wang B, Liao C, Zhou B, Cao W, Lv J, Yu C (2015). Genetic contribution to the variance of blood pressure and heart rate: a systematic review and meta-regression of twin studies. Twin Res Hum Genet.

[7] Moglia C, Calvo A, Canosa A, Bertuzzo D, Cugnasco P, Solero L (2017). Influence of arterial hypertension, type 2 diabetes and cardiovascular risk factors on ALS outcome: a population-based study. Amyotroph Lateral Scler Front Degener.

[8] Körner S, Kollewe K, Ilsemann J, Müller-Heine A, Dengler R, Krampfl K (2013). Prevalence and prognostic impact of comorbidities in amyotrophic lateral sclerosis. Eur J Neurol.

[9] Moreau C, Brunaud-Danel V, Dallongeville J, Duhamel A, Laurier-Grymonprez L, de Reuck J (2012). Modifying effect of arterial hypertension on amyotrophic lateral sclerosis. Amyotroph Lateral Scler.

[10] Hollinger SK, Okosun IS, Mitchell CS (2016). Antecedent disease and amyotrophic lateral sclerosis: what is protecting whom?. Front Neurol.

[11] Lian L, Liu M, Cui L, Guan Y, Liu T, Cui B (2019). Environmental risk factors and amyotrophic lateral sclerosis (ALS): a case-control study of ALS in China. J Clin Neurosci.

[12] Smith GD, Ebrahim S (2003). 'Mendelian randomization': can genetic epidemiology contribute to understanding environmental determinants of disease?. Int J Epidemiol.

[13] Haycock PC, Burgess S, Wade KH, Bowden J, Relton C, Davey SG (2016). Best (but oft-forgotten) practices: the design, analysis, and interpretation of Mendelian randomization studies. Am J Clin Nutr.

[14] Disney-Hogg L, Cornish AJ, Sud A, Law PJ, Kinnersley B, Jacobs DI (2018). Impact of atopy on risk of glioma: a Mendelian randomisation study. BMC Med.

[15] Evangelou E, Warren HR, Mosen-Ansorena D, Mifsud B, Pazoki R, Gao H (2018). Genetic analysis of over 1 million people identifies 535 new loci associated with blood pressure traits. Nat Genet.

[16] Nicolas A, Kenna KP, Renton AE, Ticozzi N, Faghri F, Chia R (2018). Genome-wide analyses identify KIF5A as a novel ALS gene. Neuron.

[17] Ou YN, Yang YX, Shen XN, Ma YH, Chen SD, Dong Q (2021). Genetically determined blood pressure, antihypertensive medications, and risk of Alzheimer's disease: a Mendelian randomization study. Alzheimers Res Ther.

[18] Emdin CA, Khera AV, Natarajan P, Klarin D, Zekavat SM, Hsiao AJ (2017). Genetic association of waist-to-hip ratio with cardiometabolic traits, type 2 diabetes, and coronary heart disease. JAMA.

[19] Burgess S, Bowden J, Fall T, Ingelsson E, Thompson SG (2017). Sensitivity analyses for robust causal inference from Mendelian randomization analyses with multiple genetic variants. Epidemiology.

[20] Bowden J, Davey Smith G, Haycock PC, Burgess S (2016). Consistent estimation in Mendelian randomization with some invalid instruments using a weighted median estimator. Genet Epidemiol.

[21] Burgess S, Thompson SG (2017). Interpreting findings from Mendelian randomization using the MR-Egger method. Eur J Epidemiol.

[22] Verbanck M, Chen CY, Neale B, Do R (2018). Detection of widespread horizontal pleiotropy in causal relationships inferred from Mendelian randomization between complex traits and diseases. Nat Genet.

[23] Hemani G, Zheng J, Elsworth B, Wade KH, Haberland V, Baird D (2018). The MR-Base platform supports systematic causal inference across the human phenome. eLife.

[24] Kandinov B, Drory VE, Tordjman K, Korczyn AD (2012). Blood pressure measurements in a transgenic SOD1-G93A mouse model of amyotrophic lateral sclerosis. Amyotroph Lateral Scler.

[25] Bandres-Ciga S, Noyce AJ, Hemani G, Nicolas A, Calvo A, Mora G (2019). Shared polygenic risk and causal inferences in amyotrophic lateral sclerosis. Ann Neurol.

[26] Whelton PK, Carey RM, Aronow WS, Casey DE, Collins KJ, Dennison Himmelfarb C (2018). 2017 ACC/AHA/AAPA/ABC/ACPM/AGS/APhA/ASH/ASPC/NMA/PCNA guideline for the prevention, detection, evaluation, and management of high blood pressure in adults: a report of the American College of Cardiology/American Heart Association Task Force on Clinical Practice Guidelines. Hypertension.

[27] Flint AC, Conell C, Ren X, Banki NM, Chan SL, Rao VA (2019). Effect of systolic and diastolic blood pressure on cardiovascular outcomes. N Engl J Med.

[28] Lin F-C, Tsai C-P, Kuang-Wu Lee J, Wu M-T, Tzu-Chi LC (2015). Angiotensin-converting enzyme inhibitors and amyotrophic lateral sclerosis risk: a total population-based case-control study. JAMA Neurol.

[29] Lee K, Lim C-Y (2019). Mendelian randomization analysis in observational epidemiology. J Lipid Atheroscler.

[30] Benatar M, Turner MR, Wuu J (2019). Defining pre-symptomatic amyotrophic lateral sclerosis. Amyotroph Lateral Scler Front Degener.

[31] Glodzik L, Rusinek H, Tsui W, Pirraglia E, Kim HJ, Deshpande A (2019). Different relationship between systolic blood pressure and cerebral perfusion in subjects with and without hypertension. Hypertension.

[32] Qiu C, von Strauss E, Fastbom J, Winblad B, Fratiglioni L (2003). Low blood pressure and risk of dementia in the Kungsholmen project: a 6-year follow-up study. Arch Neurol.

[33] Daulatzai MA (2017). Cerebral hypoperfusion and glucose hypometabolism: key pathophysiological modulators promote neurodegeneration, cognitive impairment, and Alzheimer's disease. J Neurosci Res.

[34] Muhire G, Iulita MF, Vallerand D, Youwakim J, Gratuze M, Petry FR (2019). Arterial stiffness due to carotid calcification disrupts cerebral blood flow regulation and leads to cognitive deficits. J Am Heart Assoc.

[35] Velebit J, Horvat A, Smolič T, Prpar Mihevc S, Rogelj B, Zorec R (2020). Astrocytes with TDP-43 inclusions exhibit reduced noradrenergic cAMP and Ca(2+) signaling and dysregulated cell metabolism. Sci Rep.

[36] Dalla Vecchia L, De Maria B, Marinou K, Sideri R, Lucini A, Porta A (2015). Cardiovascular neural regulation is impaired in amyotrophic lateral sclerosis patients. A study by spectral and complexity analysis of cardiovascular oscillations. Physiol Meas.

[37] Pimentel RMM, Macedo H, Valenti VE, Rocha FO, Abreu LC, Monteiro CBM (2019). Decreased heart rate variability in individuals with amyotrophic lateral sclerosis. Respir Care.

[38] Keskin I, Forsgren E, Lehmann M, Andersen PM, Brännström T, Lange DJ (2019). The molecular pathogenesis of superoxide dismutase 1-linked ALS is promoted by low oxygen tension. Acta Neuropathol.

[39] Bursch F, Kalmbach N, Naujock M, Staege S, Eggenschwiler R, Abo-Rady M (2019). Altered calcium dynamics and glutamate receptor properties in iPSC-derived motor neurons from ALS patients with C9orf72, FUS, SOD1 or TDP43 mutations. Hum Mol Genet.

[40] Tedeschi V, Petrozziello T, Sisalli MJ, Boscia F, Canzoniero LMT, Secondo A (2019). The activation of Mucolipin TRP channel 1 (TRPML1) protects motor neurons from L-BMAA neurotoxicity by promoting autophagic clearance. Sci Rep.

[41] Tedeschi V, Petrozziello T, Secondo A (2019). Calcium dyshomeostasis and lysosomal Ca^2+^ dysfunction in amyotrophic lateral sclerosis. Cells.

[42] Davey Smith G, Hemani G (2014). Mendelian randomization: genetic anchors for causal inference in epidemiological studies. Hum Mol Genet.

