# Supplementary material for: Assessing the role of blood pressure in amyotrophic lateral sclerosis: a Mendelian randomization study
Source: Orphanet J Rare Dis. 2022 Feb 16;17:56. doi: 10.1186/s13023-022-02212-0 (PMC8848798; doi:10.1186/s13023-022-02212-0)
Supplement: Supplementary file 1 — Additional file 1: Supplementary Table 1. Information of Instrumental Variables. [file 13023_2022_2212_MOESM1_ESM.docx]

Additional file 1

Kailin Xia ^ab^, Linjing Zhang ^ab^, Lu Tang ^ab^, Tao Huang*^de^, Dongsheng Fan*^abc^

a Department of Neurology, Peking University Third Hospital, Beijing, China.

b Beijing Municipal Key Laboratory of Biomarker and Translational Research in Neurodegenerative Diseases, Beijing, China.

c Key Laboratory for Neuroscience, National Health Commission/Ministry of Education, Peking University, Beijing, China.

d Department of Epidemiology and Biostatistics, School of Public Health, Peking University, Beijing, China.

e Key Laboratory of Molecular Cardiovascular Sciences (Peking University), Ministry of Education, Beijing, China.

Corresponding author:

Tao Huang, E-Mail: [huangtao@bjmu.edu.cn](mailto:huangtao@bjmu.edu.cn)

Dongsheng Fan, E-Mail: [dsfan2010@aliyun.com](mailto:dsfan2010@aliyun.com), ORCID: 0000-0002-3129-9821

Address: The Garden North Road No.49, 100191, Beijing, China

*These authors contributed to this work equally

| Supplementary Table1. Information of Instrumental Variables | | | | | | | | | | |
| --- | --- | --- | --- | --- | --- | --- | --- | --- | --- | --- |
| SNP | effect | standard error | p value | effect allele_freq | effect allele | the other allele | samplesize | F statistics | R^2^ | Traits |
| rs488834 | -0.3799 | 0.0365 | 2.35E-25 | 0.2355 | T | C | 757601 | 1.08E+02 | 1.43E-04 | SBP |
| rs10776752 | 0.8211 | 0.0576 | 4.61E-46 | 0.9191 | T | G | 757601 | 2.03E+02 | 2.68E-04 | SBP |
| rs59980837 | 1.0997 | 0.1163 | 3.32E-21 | 0.9822 | T | G | 757601 | 8.94E+01 | 1.18E-04 | SBP |
| rs76719272 | -0.2738 | 0.0461 | 2.97E-09 | 0.8688 | T | C | 757601 | 3.53E+01 | 4.66E-05 | SBP |
| rs75461554 | -0.3016 | 0.0377 | 1.18E-15 | 0.7993 | T | C | 757601 | 6.40E+01 | 8.45E-05 | SBP |
| rs1889785 | 0.1782 | 0.0304 | 4.35E-09 | 0.5448 | A | G | 757601 | 3.44E+01 | 4.54E-05 | SBP |
| rs12731646 | -0.189 | 0.0307 | 7.21E-10 | 0.591 | T | C | 757601 | 3.79E+01 | 5.00E-05 | SBP |
| rs1043069 | 0.234 | 0.0311 | 5.26E-14 | 0.3844 | T | G | 757601 | 5.66E+01 | 7.47E-05 | SBP |
| rs4651224 | 0.1986 | 0.0306 | 9.00E-11 | 0.5526 | T | C | 757601 | 4.21E+01 | 5.56E-05 | SBP |
| rs12042924 | -0.1807 | 0.0303 | 2.62E-09 | 0.4716 | T | C | 757601 | 3.56E+01 | 4.69E-05 | SBP |
| rs11120093 | -0.1792 | 0.0307 | 5.13E-09 | 0.5918 | T | C | 757601 | 3.41E+01 | 4.50E-05 | SBP |
| rs2724377 | 0.1938 | 0.0301 | 1.29E-10 | 0.4697 | A | G | 757601 | 4.15E+01 | 5.47E-05 | SBP |
| rs263532 | 0.1798 | 0.0307 | 4.72E-09 | 0.4245 | T | C | 757601 | 3.43E+01 | 4.53E-05 | SBP |
| rs68085857 | 0.274 | 0.0357 | 1.68E-14 | 0.766 | T | C | 757601 | 5.89E+01 | 7.77E-05 | SBP |
| rs4595370 | -0.2092 | 0.0328 | 1.73E-10 | 0.6988 | A | G | 757601 | 4.07E+01 | 5.37E-05 | SBP |
| rs1745417 | 0.2871 | 0.0301 | 1.59E-21 | 0.4799 | T | C | 757601 | 9.10E+01 | 1.20E-04 | SBP |
| rs699 | -0.3748 | 0.0308 | 5.59E-34 | 0.4072 | A | G | 757601 | 1.48E+02 | 1.95E-04 | SBP |
| rs1565440 | 0.1746 | 0.0311 | 1.94E-08 | 0.6248 | A | G | 757601 | 3.15E+01 | 4.16E-05 | SBP |
| rs404100 | 0.1935 | 0.0303 | 1.68E-10 | 0.5487 | T | C | 757601 | 4.08E+01 | 5.38E-05 | SBP |
| rs34079867 | 0.1992 | 0.0354 | 1.78E-08 | 0.734 | T | C | 757601 | 3.17E+01 | 4.18E-05 | SBP |
| rs4908348 | 0.2366 | 0.033 | 8.07E-13 | 0.3056 | T | G | 757601 | 5.14E+01 | 6.78E-05 | SBP |
| rs2493296 | 0.4183 | 0.0442 | 3.14E-21 | 0.8575 | T | C | 757601 | 8.96E+01 | 1.18E-04 | SBP |
| rs11210029 | -0.203 | 0.0313 | 8.92E-11 | 0.3678 | A | G | 757601 | 4.21E+01 | 5.55E-05 | SBP |
| rs1408945 | -0.3196 | 0.0304 | 8.33E-26 | 0.5757 | T | G | 757601 | 1.11E+02 | 1.46E-04 | SBP |
| rs1209384 | 0.2558 | 0.0313 | 2.85E-16 | 0.6122 | A | G | 757601 | 6.68E+01 | 8.82E-05 | SBP |
| rs778124 | 0.2965 | 0.0311 | 1.45E-21 | 0.6264 | A | G | 757601 | 9.09E+01 | 1.20E-04 | SBP |
| rs61772592 | -0.3181 | 0.0455 | 2.86E-12 | 0.1255 | A | G | 757601 | 4.89E+01 | 6.45E-05 | SBP |
| rs12063372 | 0.1989 | 0.0318 | 3.86E-10 | 0.6154 | A | G | 757601 | 3.91E+01 | 5.16E-05 | SBP |
| rs2232460 | -0.2171 | 0.032 | 1.10E-11 | 0.6657 | A | G | 757601 | 4.60E+01 | 6.08E-05 | SBP |
| rs12136922 | 0.2027 | 0.0304 | 2.69E-11 | 0.5051 | A | G | 757601 | 4.45E+01 | 5.87E-05 | SBP |
| rs658780 | -0.2028 | 0.0347 | 5.29E-09 | 0.2553 | T | G | 757601 | 3.42E+01 | 4.51E-05 | SBP |
| rs786923 | -0.3082 | 0.031 | 2.83E-23 | 0.3761 | T | C | 757601 | 9.88E+01 | 1.30E-04 | SBP |
| rs7514579 | 0.2243 | 0.0361 | 5.45E-10 | 0.2288 | A | C | 757601 | 3.86E+01 | 5.10E-05 | SBP |
| rs1006545 | 0.6846 | 0.048 | 3.50E-46 | 0.1128 | T | G | 757601 | 2.03E+02 | 2.68E-04 | SBP |
| rs11191580 | 1.0995 | 0.055 | 7.74E-89 | 0.0824 | T | C | 757601 | 4.00E+02 | 5.27E-04 | SBP |
| rs117464403 | 0.864 | 0.1199 | 5.80E-13 | 0.9817 | A | G | 757601 | 5.19E+01 | 6.85E-05 | SBP |
| rs12255372 | 0.2358 | 0.0335 | 1.94E-12 | 0.7117 | T | G | 757601 | 4.95E+01 | 6.54E-05 | SBP |
| rs72842207 | -0.203 | 0.0367 | 3.14E-08 | 0.7856 | T | C | 757601 | 3.06E+01 | 4.04E-05 | SBP |
| rs11592107 | 0.3024 | 0.0326 | 1.55E-20 | 0.6904 | A | G | 757601 | 8.60E+01 | 1.14E-04 | SBP |
| rs7093894 | 0.236 | 0.0427 | 3.16E-08 | 0.8488 | A | C | 757601 | 3.05E+01 | 4.03E-05 | SBP |
| rs7912283 | -0.2144 | 0.0322 | 2.94E-11 | 0.3532 | A | G | 757601 | 4.43E+01 | 5.85E-05 | SBP |
| rs1133400 | -0.2975 | 0.0376 | 2.53E-15 | 0.214 | A | G | 757601 | 6.26E+01 | 8.26E-05 | SBP |
| rs1623474 | 0.3827 | 0.0321 | 7.66E-33 | 0.6697 | T | C | 757601 | 1.42E+02 | 1.88E-04 | SBP |
| rs12264186 | 0.2135 | 0.0387 | 3.58E-08 | 0.8129 | T | C | 757601 | 3.04E+01 | 4.02E-05 | SBP |
| rs11252324 | -0.4164 | 0.0573 | 3.61E-13 | 0.9229 | T | G | 757601 | 5.28E+01 | 6.97E-05 | SBP |
| rs4948643 | 0.2258 | 0.0338 | 2.40E-11 | 0.7181 | T | C | 757601 | 4.46E+01 | 5.89E-05 | SBP |
| rs34130368 | -0.3016 | 0.0497 | 1.28E-09 | 0.883 | T | G | 757601 | 3.68E+01 | 4.86E-05 | SBP |
| rs4245599 | -0.1794 | 0.0305 | 4.04E-09 | 0.5416 | A | G | 757601 | 3.46E+01 | 4.57E-05 | SBP |
| rs57946343 | 0.716 | 0.0426 | 2.10E-63 | 0.1473 | T | C | 757601 | 2.82E+02 | 3.73E-04 | SBP |
| rs2236295 | -0.3028 | 0.0309 | 1.05E-22 | 0.6022 | T | G | 757601 | 9.60E+01 | 1.27E-04 | SBP |
| rs2177843 | 0.4394 | 0.0432 | 2.80E-24 | 0.8495 | T | C | 757601 | 1.03E+02 | 1.37E-04 | SBP |
| rs10749572 | -0.203 | 0.0302 | 1.88E-11 | 0.4556 | T | G | 757601 | 4.52E+01 | 5.96E-05 | SBP |
| rs111866816 | 0.3569 | 0.0597 | 2.29E-09 | 0.9291 | T | C | 757601 | 3.57E+01 | 4.72E-05 | SBP |
| rs2689690 | -0.2702 | 0.0316 | 1.15E-17 | 0.6322 | T | C | 757601 | 7.31E+01 | 9.65E-05 | SBP |
| rs604723 | -0.655 | 0.0339 | 2.55E-83 | 0.7244 | T | C | 757601 | 3.73E+02 | 4.93E-04 | SBP |
| rs629864 | -0.1868 | 0.0319 | 4.69E-09 | 0.3503 | T | C | 757601 | 3.43E+01 | 4.53E-05 | SBP |
| rs7926110 | 0.2603 | 0.0321 | 5.71E-16 | 0.3267 | T | G | 757601 | 6.58E+01 | 8.68E-05 | SBP |
| rs236916 | 0.3166 | 0.0446 | 1.31E-12 | 0.8652 | A | G | 757601 | 5.04E+01 | 6.65E-05 | SBP |
| rs573455 | 0.1994 | 0.0303 | 4.77E-11 | 0.539 | A | G | 757601 | 4.33E+01 | 5.72E-05 | SBP |
| rs7944927 | 0.2235 | 0.0392 | 1.23E-08 | 0.2181 | T | C | 757601 | 3.25E+01 | 4.29E-05 | SBP |
| rs2014408 | 0.5169 | 0.0373 | 1.26E-43 | 0.7913 | T | C | 757601 | 1.92E+02 | 2.53E-04 | SBP |
| rs7926335 | 0.3135 | 0.0339 | 2.52E-20 | 0.7309 | T | C | 757601 | 8.55E+01 | 1.13E-04 | SBP |
| rs569550 | -0.5765 | 0.0318 | 1.33E-73 | 0.3963 | T | G | 757601 | 3.29E+02 | 4.34E-04 | SBP |
| rs74048190 | -0.4404 | 0.0757 | 6.07E-09 | 0.0478 | T | C | 757601 | 3.38E+01 | 4.47E-05 | SBP |
| rs17762 | 0.4117 | 0.0571 | 5.60E-13 | 0.9223 | A | G | 757601 | 5.20E+01 | 6.86E-05 | SBP |
| rs1382472 | -0.1917 | 0.0307 | 4.47E-10 | 0.5959 | A | G | 757601 | 3.90E+01 | 5.15E-05 | SBP |
| rs871004 | 0.2336 | 0.0317 | 1.65E-13 | 0.6519 | A | G | 757601 | 5.43E+01 | 7.17E-05 | SBP |
| rs10501122 | 0.1916 | 0.0315 | 1.18E-09 | 0.361 | T | C | 757601 | 3.70E+01 | 4.88E-05 | SBP |
| rs11604310 | -0.2778 | 0.0411 | 1.46E-11 | 0.8345 | T | C | 757601 | 4.57E+01 | 6.03E-05 | SBP |
| rs7107356 | -0.4598 | 0.0301 | 1.63E-52 | 0.5041 | A | G | 757601 | 2.33E+02 | 3.08E-04 | SBP |
| rs2904315 | -0.2081 | 0.0325 | 1.58E-10 | 0.6869 | A | G | 757601 | 4.10E+01 | 5.41E-05 | SBP |
| rs4427587 | 0.2062 | 0.0313 | 4.28E-11 | 0.4381 | T | C | 757601 | 4.34E+01 | 5.73E-05 | SBP |
| rs7125196 | 0.4422 | 0.0472 | 7.31E-21 | 0.1183 | T | C | 757601 | 8.78E+01 | 1.16E-04 | SBP |
| rs2306363 | -0.4358 | 0.0376 | 5.24E-31 | 0.7955 | T | G | 757601 | 1.34E+02 | 1.77E-04 | SBP |
| rs7395791 | -0.2162 | 0.0308 | 2.19E-12 | 0.5581 | A | G | 757601 | 4.93E+01 | 6.50E-05 | SBP |
| rs10501410 | 0.4122 | 0.0607 | 1.10E-11 | 0.9308 | A | G | 757601 | 4.61E+01 | 6.09E-05 | SBP |
| rs7927515 | 0.2271 | 0.0319 | 1.05E-12 | 0.6541 | A | C | 757601 | 5.07E+01 | 6.69E-05 | SBP |
| rs2289124 | -0.308 | 0.0415 | 1.14E-13 | 0.8327 | A | G | 757601 | 5.51E+01 | 7.27E-05 | SBP |
| rs360153 | -0.3445 | 0.0306 | 1.73E-29 | 0.5834 | T | C | 757601 | 1.27E+02 | 1.67E-04 | SBP |
| rs5742643 | -0.2233 | 0.0349 | 1.53E-10 | 0.7513 | T | C | 757601 | 4.09E+01 | 5.40E-05 | SBP |
| rs1896326 | -0.2797 | 0.0371 | 4.41E-14 | 0.7709 | A | G | 757601 | 5.68E+01 | 7.50E-05 | SBP |
| rs35444 | 0.4368 | 0.031 | 3.47E-45 | 0.3862 | A | G | 757601 | 1.99E+02 | 2.62E-04 | SBP |
| rs6490019 | -0.2897 | 0.0309 | 6.61E-21 | 0.6204 | A | G | 757601 | 8.79E+01 | 1.16E-04 | SBP |
| rs117206641 | 0.3154 | 0.0499 | 2.66E-10 | 0.8892 | T | C | 757601 | 4.00E+01 | 5.27E-05 | SBP |
| rs1010064 | 0.3571 | 0.0387 | 3.02E-20 | 0.1837 | A | C | 757601 | 8.51E+01 | 1.12E-04 | SBP |
| rs73075659 | 0.3962 | 0.0321 | 5.52E-35 | 0.3346 | A | G | 757601 | 1.52E+02 | 2.01E-04 | SBP |
| rs3819532 | -0.1875 | 0.0306 | 9.44E-10 | 0.6087 | T | C | 757601 | 3.75E+01 | 4.96E-05 | SBP |
| rs9651825 | -0.2042 | 0.034 | 1.93E-09 | 0.2705 | A | G | 757601 | 3.61E+01 | 4.76E-05 | SBP |
| rs61917655 | 0.3427 | 0.0514 | 2.68E-11 | 0.8986 | T | C | 757601 | 4.45E+01 | 5.87E-05 | SBP |
| rs12426261 | 0.3775 | 0.0309 | 2.31E-34 | 0.6208 | A | G | 757601 | 1.49E+02 | 1.97E-04 | SBP |
| rs7134440 | 0.4788 | 0.0562 | 1.58E-17 | 0.9178 | T | C | 757601 | 7.26E+01 | 9.58E-05 | SBP |
| rs7134677 | -0.3851 | 0.0332 | 4.46E-31 | 0.7022 | T | C | 757601 | 1.35E+02 | 1.78E-04 | SBP |
| rs7306710 | -0.2429 | 0.0303 | 1.03E-15 | 0.519 | T | C | 757601 | 6.43E+01 | 8.48E-05 | SBP |
| rs4143175 | 0.2187 | 0.0352 | 5.10E-10 | 0.7591 | T | C | 757601 | 3.86E+01 | 5.10E-05 | SBP |
| rs7963801 | -0.2362 | 0.0311 | 2.87E-14 | 0.5779 | T | C | 757601 | 5.77E+01 | 7.61E-05 | SBP |
| rs6539467 | 0.265 | 0.0404 | 5.57E-11 | 0.8339 | A | G | 757601 | 4.30E+01 | 5.68E-05 | SBP |
| rs17249754 | -0.8446 | 0.0403 | 1.25E-97 | 0.8317 | A | G | 757601 | 4.39E+02 | 5.79E-04 | SBP |
| rs10777213 | -0.1786 | 0.0299 | 2.45E-09 | 0.4756 | A | G | 757601 | 3.57E+01 | 4.71E-05 | SBP |
| rs9549627 | 0.2846 | 0.05 | 1.25E-08 | 0.8825 | A | G | 757601 | 3.24E+01 | 4.28E-05 | SBP |
| rs7331680 | 0.4101 | 0.0423 | 3.35E-22 | 0.8509 | T | G | 757601 | 9.40E+01 | 1.24E-04 | SBP |
| rs483071 | 0.2709 | 0.0313 | 5.09E-18 | 0.3752 | T | C | 757601 | 7.49E+01 | 9.89E-05 | SBP |
| rs9507885 | -0.3208 | 0.0542 | 3.23E-09 | 0.9047 | T | C | 757601 | 3.50E+01 | 4.62E-05 | SBP |
| rs2065498 | -0.2934 | 0.0403 | 3.36E-13 | 0.8294 | T | G | 757601 | 5.30E+01 | 7.00E-05 | SBP |
| rs7491248 | 0.2163 | 0.0362 | 2.38E-09 | 0.7761 | A | G | 757601 | 3.57E+01 | 4.71E-05 | SBP |
| rs9526707 | -0.2039 | 0.0323 | 2.77E-10 | 0.6784 | A | G | 757601 | 3.99E+01 | 5.26E-05 | SBP |
| rs75961402 | 0.2659 | 0.0418 | 1.95E-10 | 0.8466 | A | G | 757601 | 4.05E+01 | 5.34E-05 | SBP |
| rs17245822 | -0.1899 | 0.0312 | 1.15E-09 | 0.3733 | A | C | 757601 | 3.70E+01 | 4.89E-05 | SBP |
| rs78474310 | -0.4699 | 0.0734 | 1.51E-10 | 0.0448 | A | G | 757601 | 4.10E+01 | 5.41E-05 | SBP |
| rs6562778 | 0.178 | 0.0304 | 4.96E-09 | 0.5411 | A | G | 757601 | 3.43E+01 | 4.53E-05 | SBP |
| rs17562391 | 0.1967 | 0.0306 | 1.35E-10 | 0.5814 | T | C | 757601 | 4.13E+01 | 5.45E-05 | SBP |
| rs75016974 | -0.2513 | 0.0439 | 1.05E-08 | 0.8577 | T | C | 757601 | 3.28E+01 | 4.33E-05 | SBP |
| rs12885878 | -0.2291 | 0.0367 | 4.32E-10 | 0.7663 | A | G | 757601 | 3.90E+01 | 5.14E-05 | SBP |
| rs365990 | 0.225 | 0.0312 | 5.95E-13 | 0.3658 | A | G | 757601 | 5.20E+01 | 6.86E-05 | SBP |
| rs8904 | 0.3061 | 0.0314 | 1.71E-22 | 0.6322 | A | G | 757601 | 9.50E+01 | 1.25E-04 | SBP |
| rs72683923 | 0.9587 | 0.1101 | 3.08E-18 | 0.0212 | T | C | 757601 | 7.58E+01 | 1.00E-04 | SBP |
| rs35413927 | -0.3002 | 0.0328 | 5.25E-20 | 0.3054 | A | G | 757601 | 8.38E+01 | 1.11E-04 | SBP |
| rs11159091 | 0.1978 | 0.0303 | 6.79E-11 | 0.5385 | A | G | 757601 | 4.26E+01 | 5.62E-05 | SBP |
| rs7154723 | 0.253 | 0.0309 | 2.72E-16 | 0.615 | A | G | 757601 | 6.70E+01 | 8.85E-05 | SBP |
| rs4606697 | -0.3196 | 0.0523 | 9.71E-10 | 0.8959 | A | G | 757601 | 3.73E+01 | 4.93E-05 | SBP |
| rs28866311 | -0.2762 | 0.0302 | 5.45E-20 | 0.4737 | T | G | 757601 | 8.36E+01 | 1.10E-04 | SBP |
| rs4775769 | -0.4162 | 0.0517 | 7.76E-16 | 0.9055 | T | G | 757601 | 6.48E+01 | 8.55E-05 | SBP |
| rs3098186 | -0.2422 | 0.0303 | 1.41E-15 | 0.4844 | T | C | 757601 | 6.39E+01 | 8.43E-05 | SBP |
| rs2652812 | -0.2516 | 0.0353 | 1.03E-12 | 0.2456 | T | C | 757601 | 5.08E+01 | 6.71E-05 | SBP |
| rs28429256 | 0.215 | 0.0325 | 3.89E-11 | 0.6658 | A | G | 757601 | 4.38E+01 | 5.78E-05 | SBP |
| rs11636952 | 0.5313 | 0.0328 | 4.22E-59 | 0.6859 | T | C | 757601 | 2.62E+02 | 3.46E-04 | SBP |
| rs2627313 | 0.3208 | 0.0303 | 3.55E-26 | 0.5546 | T | C | 757601 | 1.12E+02 | 1.48E-04 | SBP |
| rs2046341 | -0.2542 | 0.0382 | 2.74E-11 | 0.8079 | A | G | 757601 | 4.43E+01 | 5.84E-05 | SBP |
| rs77032376 | -0.2727 | 0.043 | 2.35E-10 | 0.8515 | T | C | 757601 | 4.02E+01 | 5.31E-05 | SBP |
| rs4932373 | -0.635 | 0.0328 | 2.49E-83 | 0.3258 | A | C | 757601 | 3.75E+02 | 4.94E-04 | SBP |
| rs12906962 | -0.2653 | 0.0325 | 3.28E-16 | 0.324 | T | C | 757601 | 6.66E+01 | 8.79E-05 | SBP |
| rs2589218 | -0.2258 | 0.0339 | 2.54E-11 | 0.2703 | T | C | 757601 | 4.44E+01 | 5.86E-05 | SBP |
| rs11641374 | -0.1943 | 0.0309 | 3.26E-10 | 0.4005 | A | C | 757601 | 3.95E+01 | 5.22E-05 | SBP |
| rs77924615 | -0.4081 | 0.039 | 1.12E-25 | 0.8014 | A | G | 757601 | 1.09E+02 | 1.45E-04 | SBP |
| rs12596630 | 0.4278 | 0.0547 | 5.01E-15 | 0.9097 | T | C | 757601 | 6.12E+01 | 8.07E-05 | SBP |
| rs7186298 | -0.2315 | 0.0302 | 1.88E-14 | 0.5705 | T | C | 757601 | 5.88E+01 | 7.76E-05 | SBP |
| rs8044992 | 0.2138 | 0.0331 | 1.07E-10 | 0.2877 | T | C | 757601 | 4.17E+01 | 5.51E-05 | SBP |
| rs72778133 | -0.2417 | 0.0443 | 4.98E-08 | 0.1422 | T | C | 757601 | 2.98E+01 | 3.93E-05 | SBP |
| rs111929315 | 0.3146 | 0.0485 | 8.60E-11 | 0.1083 | A | G | 757601 | 4.21E+01 | 5.55E-05 | SBP |
| rs12446456 | -0.3003 | 0.0302 | 2.97E-23 | 0.5726 | T | C | 757601 | 9.89E+01 | 1.30E-04 | SBP |
| rs34941092 | -0.3225 | 0.0425 | 3.23E-14 | 0.8502 | A | G | 757601 | 5.76E+01 | 7.60E-05 | SBP |
| rs4784541 | -0.2015 | 0.0307 | 4.93E-11 | 0.5252 | T | C | 757601 | 4.31E+01 | 5.69E-05 | SBP |
| rs2060664 | 0.216 | 0.0345 | 4.06E-10 | 0.2516 | T | C | 757601 | 3.92E+01 | 5.17E-05 | SBP |
| rs146550789 | -0.4824 | 0.0778 | 5.64E-10 | 0.0417 | T | C | 757601 | 3.84E+01 | 5.07E-05 | SBP |
| rs62047964 | 0.5115 | 0.0686 | 9.29E-14 | 0.9378 | T | C | 757601 | 5.56E+01 | 7.34E-05 | SBP |
| rs4888408 | 0.3653 | 0.0307 | 1.42E-32 | 0.4145 | A | G | 757601 | 1.42E+02 | 1.87E-04 | SBP |
| rs12926550 | -0.2548 | 0.0324 | 3.43E-15 | 0.6844 | A | G | 757601 | 6.18E+01 | 8.16E-05 | SBP |
| rs3950627 | 0.1851 | 0.0308 | 1.82E-09 | 0.469 | A | C | 757601 | 3.61E+01 | 4.77E-05 | SBP |
| rs908951 | -0.2261 | 0.0315 | 7.14E-13 | 0.5622 | T | C | 757601 | 5.15E+01 | 6.80E-05 | SBP |
| rs9303175 | -0.2048 | 0.0327 | 3.65E-10 | 0.6537 | T | G | 757601 | 3.92E+01 | 5.18E-05 | SBP |
| rs4925159 | 0.2174 | 0.0305 | 9.66E-13 | 0.5754 | A | G | 757601 | 5.08E+01 | 6.71E-05 | SBP |
| rs7218708 | -0.1781 | 0.0303 | 4.38E-09 | 0.5169 | A | G | 757601 | 3.45E+01 | 4.56E-05 | SBP |
| rs11653927 | -0.2796 | 0.0308 | 1.17E-19 | 0.6155 | T | C | 757601 | 8.24E+01 | 1.09E-04 | SBP |
| rs1551355 | 0.2098 | 0.0356 | 3.89E-09 | 0.7666 | T | C | 757601 | 3.47E+01 | 4.58E-05 | SBP |
| rs7213273 | -0.4 | 0.0315 | 6.24E-37 | 0.345 | A | G | 757601 | 1.61E+02 | 2.13E-04 | SBP |
| rs17608766 | -0.6903 | 0.0433 | 2.48E-57 | 0.1445 | T | C | 757601 | 2.54E+02 | 3.35E-04 | SBP |
| rs3764400 | 0.3748 | 0.0445 | 3.69E-17 | 0.1365 | T | C | 757601 | 7.09E+01 | 9.36E-05 | SBP |
| rs9897429 | 0.2645 | 0.0319 | 1.19E-16 | 0.48 | A | G | 757601 | 6.87E+01 | 9.07E-05 | SBP |
| rs1000423 | 0.4138 | 0.0346 | 6.50E-33 | 0.2684 | T | C | 757601 | 1.43E+02 | 1.89E-04 | SBP |
| rs56288724 | -0.2178 | 0.031 | 2.01E-12 | 0.4169 | A | G | 757601 | 4.94E+01 | 6.52E-05 | SBP |
| rs62076622 | 0.2363 | 0.0377 | 3.79E-10 | 0.1987 | A | G | 757601 | 3.93E+01 | 5.19E-05 | SBP |
| rs6504213 | -0.2982 | 0.0312 | 1.25E-21 | 0.5818 | T | C | 757601 | 9.13E+01 | 1.21E-04 | SBP |
| rs113086489 | 0.3249 | 0.0307 | 3.80E-26 | 0.4475 | T | C | 757601 | 1.12E+02 | 1.48E-04 | SBP |
| rs4511593 | -0.2881 | 0.0318 | 1.28E-19 | 0.3472 | T | C | 757601 | 8.21E+01 | 1.08E-04 | SBP |
| rs1436138 | 0.3119 | 0.0315 | 4.73E-23 | 0.3633 | A | G | 757601 | 9.80E+01 | 1.29E-04 | SBP |
| rs9302885 | 0.2242 | 0.0302 | 1.03E-13 | 0.5548 | A | G | 757601 | 5.51E+01 | 7.27E-05 | SBP |
| rs117285318 | 0.4413 | 0.0589 | 6.93E-14 | 0.0775 | T | C | 757601 | 5.61E+01 | 7.41E-05 | SBP |
| rs11655604 | -0.2033 | 0.0333 | 1.09E-09 | 0.6421 | T | C | 757601 | 3.73E+01 | 4.92E-05 | SBP |
| rs1154214 | -0.2031 | 0.0306 | 3.27E-11 | 0.6037 | T | G | 757601 | 4.41E+01 | 5.81E-05 | SBP |
| rs56407827 | 0.3603 | 0.034 | 2.78E-26 | 0.7313 | T | C | 757601 | 1.12E+02 | 1.48E-04 | SBP |
| rs11874246 | 0.2856 | 0.0328 | 3.23E-18 | 0.7037 | T | C | 757601 | 7.58E+01 | 1.00E-04 | SBP |
| rs7245140 | -0.3367 | 0.0391 | 7.67E-18 | 0.1802 | T | C | 757601 | 7.42E+01 | 9.79E-05 | SBP |
| rs1437649 | -0.2189 | 0.0357 | 8.57E-10 | 0.7655 | A | G | 757601 | 3.76E+01 | 4.96E-05 | SBP |
| rs665445 | -0.1909 | 0.0334 | 1.15E-08 | 0.7206 | A | C | 757601 | 3.27E+01 | 4.31E-05 | SBP |
| rs10048404 | -0.2607 | 0.0317 | 1.91E-16 | 0.6299 | T | C | 757601 | 6.76E+01 | 8.93E-05 | SBP |
| rs10460108 | 0.2141 | 0.0301 | 1.12E-12 | 0.5199 | A | G | 757601 | 5.06E+01 | 6.68E-05 | SBP |
| rs167479 | -0.5642 | 0.0327 | 7.21E-67 | 0.5274 | T | G | 757601 | 2.98E+02 | 3.93E-04 | SBP |
| rs698748 | 0.1871 | 0.0325 | 8.90E-09 | 0.579 | A | G | 757601 | 3.31E+01 | 4.37E-05 | SBP |
| rs1077795 | 0.2507 | 0.0344 | 3.33E-13 | 0.2607 | A | G | 757601 | 5.31E+01 | 7.01E-05 | SBP |
| rs149339216 | -0.6912 | 0.0779 | 6.93E-19 | 0.0434 | T | C | 757601 | 7.87E+01 | 1.04E-04 | SBP |
| rs62112908 | -0.2388 | 0.0419 | 1.25E-08 | 0.1536 | A | G | 757601 | 3.25E+01 | 4.29E-05 | SBP |
| rs28572357 | -0.2733 | 0.0308 | 6.34E-19 | 0.3977 | A | C | 757601 | 7.87E+01 | 1.04E-04 | SBP |
| rs1433121 | -0.228 | 0.0326 | 2.66E-12 | 0.3094 | T | C | 757601 | 4.89E+01 | 6.46E-05 | SBP |
| rs33836 | 0.1766 | 0.0304 | 6.56E-09 | 0.5378 | T | C | 757601 | 3.37E+01 | 4.45E-05 | SBP |
| rs10420519 | -0.4921 | 0.0887 | 2.86E-08 | 0.9653 | T | G | 757601 | 3.08E+01 | 4.06E-05 | SBP |
| rs7255933 | 0.2306 | 0.0345 | 2.44E-11 | 0.7426 | A | G | 757601 | 4.47E+01 | 5.90E-05 | SBP |
| rs11672660 | 0.2212 | 0.0381 | 6.32E-09 | 0.8004 | T | C | 757601 | 3.37E+01 | 4.45E-05 | SBP |
| rs571689 | 0.228 | 0.0304 | 6.77E-14 | 0.4804 | T | C | 757601 | 5.63E+01 | 7.42E-05 | SBP |
| rs73046792 | -0.3554 | 0.0426 | 7.23E-17 | 0.8412 | A | G | 757601 | 6.96E+01 | 9.19E-05 | SBP |
| rs68096471 | -0.2098 | 0.0343 | 9.26E-10 | 0.7341 | A | G | 757601 | 3.74E+01 | 4.94E-05 | SBP |
| rs12985940 | 0.4642 | 0.0434 | 1.08E-26 | 0.1592 | T | C | 757601 | 1.14E+02 | 1.51E-04 | SBP |
| rs10207726 | -0.2142 | 0.033 | 8.06E-11 | 0.704 | T | C | 757601 | 4.21E+01 | 5.56E-05 | SBP |
| rs6737318 | 0.2348 | 0.0364 | 1.13E-10 | 0.2218 | A | G | 757601 | 4.16E+01 | 5.49E-05 | SBP |
| rs2580350 | 0.1769 | 0.0307 | 8.39E-09 | 0.4391 | A | G | 757601 | 3.32E+01 | 4.38E-05 | SBP |
| rs17257081 | 0.2274 | 0.0392 | 6.35E-09 | 0.1935 | A | G | 757601 | 3.37E+01 | 4.44E-05 | SBP |
| rs55944332 | -0.2613 | 0.0355 | 1.79E-13 | 0.2368 | A | G | 757601 | 5.42E+01 | 7.15E-05 | SBP |
| rs62170470 | 0.1972 | 0.0321 | 7.69E-10 | 0.3983 | T | C | 757601 | 3.77E+01 | 4.98E-05 | SBP |
| rs62187653 | 0.3286 | 0.0511 | 1.23E-10 | 0.0971 | T | C | 757601 | 4.14E+01 | 5.46E-05 | SBP |
| rs4667454 | 0.2636 | 0.0322 | 2.63E-16 | 0.3295 | A | G | 757601 | 6.70E+01 | 8.85E-05 | SBP |
| rs10048760 | -0.1862 | 0.0301 | 6.56E-10 | 0.4712 | T | G | 757601 | 3.83E+01 | 5.05E-05 | SBP |
| rs4894132 | 0.2469 | 0.0342 | 5.51E-13 | 0.2717 | T | C | 757601 | 5.21E+01 | 6.88E-05 | SBP |
| rs12473915 | -0.295 | 0.0375 | 3.42E-15 | 0.7983 | A | G | 757601 | 6.19E+01 | 8.17E-05 | SBP |
| rs13412750 | -0.2889 | 0.0341 | 2.33E-17 | 0.7292 | A | G | 757601 | 7.18E+01 | 9.47E-05 | SBP |
| rs17760259 | -0.2654 | 0.0304 | 2.25E-18 | 0.4276 | T | C | 757601 | 7.62E+01 | 1.01E-04 | SBP |
| rs12693982 | 0.2575 | 0.0309 | 7.49E-17 | 0.5976 | T | C | 757601 | 6.94E+01 | 9.17E-05 | SBP |
| rs12694277 | -0.2018 | 0.0335 | 1.80E-09 | 0.7054 | T | C | 757601 | 3.63E+01 | 4.79E-05 | SBP |
| rs2161967 | 0.2836 | 0.0307 | 2.87E-20 | 0.5721 | T | G | 757601 | 8.53E+01 | 1.13E-04 | SBP |
| rs10804330 | 0.2351 | 0.0306 | 1.62E-14 | 0.4332 | T | C | 757601 | 5.90E+01 | 7.79E-05 | SBP |
| rs1044822 | -0.248 | 0.0424 | 5.16E-09 | 0.8518 | T | C | 757601 | 3.42E+01 | 4.52E-05 | SBP |
| rs3754944 | 0.1768 | 0.0308 | 9.30E-09 | 0.4125 | A | C | 757601 | 3.30E+01 | 4.35E-05 | SBP |
| rs145042302 | -0.5886 | 0.0972 | 1.39E-09 | 0.97 | A | G | 757601 | 3.67E+01 | 4.84E-05 | SBP |
| rs2384063 | 0.3266 | 0.0357 | 6.33E-20 | 0.2393 | T | C | 757601 | 8.37E+01 | 1.10E-04 | SBP |
| rs1275988 | -0.541 | 0.0308 | 4.42E-69 | 0.3888 | T | C | 757601 | 3.09E+02 | 4.07E-04 | SBP |
| rs13420463 | 0.3143 | 0.036 | 2.72E-18 | 0.2266 | A | G | 757601 | 7.62E+01 | 1.01E-04 | SBP |
| rs4952609 | 0.2124 | 0.0347 | 9.60E-10 | 0.2561 | A | G | 757601 | 3.75E+01 | 4.95E-05 | SBP |
| rs12464602 | -0.2437 | 0.0315 | 1.02E-14 | 0.3792 | A | G | 757601 | 5.99E+01 | 7.90E-05 | SBP |
| rs13016772 | 0.2522 | 0.0355 | 1.23E-12 | 0.2349 | T | C | 757601 | 5.05E+01 | 6.66E-05 | SBP |
| rs2249105 | 0.2927 | 0.0313 | 7.63E-21 | 0.3679 | A | G | 757601 | 8.74E+01 | 1.15E-04 | SBP |
| rs10188003 | 0.1883 | 0.0307 | 8.80E-10 | 0.607 | T | C | 757601 | 3.76E+01 | 4.97E-05 | SBP |
| rs6731373 | 0.1913 | 0.0326 | 4.18E-09 | 0.6508 | A | G | 757601 | 3.44E+01 | 4.55E-05 | SBP |
| rs4577304 | -0.1767 | 0.0302 | 4.99E-09 | 0.4767 | T | C | 757601 | 3.42E+01 | 4.52E-05 | SBP |
| rs72847885 | 0.2413 | 0.0318 | 3.08E-14 | 0.337 | A | G | 757601 | 5.76E+01 | 7.60E-05 | SBP |
| rs2423514 | 0.3011 | 0.0302 | 1.77E-23 | 0.4589 | A | G | 757601 | 9.94E+01 | 1.31E-04 | SBP |
| rs6108787 | -0.4274 | 0.03 | 5.38E-46 | 0.4704 | T | G | 757601 | 2.03E+02 | 2.68E-04 | SBP |
| rs6078093 | -0.1849 | 0.0304 | 1.20E-09 | 0.572 | A | G | 757601 | 3.70E+01 | 4.88E-05 | SBP |
| rs8125763 | 0.1761 | 0.0301 | 4.84E-09 | 0.5283 | A | C | 757601 | 3.42E+01 | 4.52E-05 | SBP |
| rs17812022 | -0.3613 | 0.0525 | 5.65E-12 | 0.9042 | T | C | 757601 | 4.74E+01 | 6.25E-05 | SBP |
| rs6058088 | 0.2832 | 0.0417 | 1.14E-11 | 0.1561 | T | G | 757601 | 4.61E+01 | 6.09E-05 | SBP |
| rs79384779 | 0.3179 | 0.0428 | 1.08E-13 | 0.8488 | T | C | 757601 | 5.52E+01 | 7.28E-05 | SBP |
| rs6029756 | -0.2712 | 0.033 | 1.88E-16 | 0.6775 | A | G | 757601 | 6.75E+01 | 8.91E-05 | SBP |
| rs6031431 | -0.2617 | 0.0304 | 7.05E-18 | 0.4624 | A | G | 757601 | 7.41E+01 | 9.78E-05 | SBP |
| rs2598 | 0.168 | 0.0303 | 2.87E-08 | 0.467 | A | G | 757601 | 3.07E+01 | 4.06E-05 | SBP |
| rs6090907 | -0.3854 | 0.0425 | 1.29E-19 | 0.853 | A | G | 757601 | 8.22E+01 | 1.09E-04 | SBP |
| rs234623 | -0.1804 | 0.0302 | 2.43E-09 | 0.4959 | A | G | 757601 | 3.57E+01 | 4.71E-05 | SBP |
| rs28374392 | 0.1924 | 0.0338 | 1.21E-08 | 0.3769 | T | C | 757601 | 3.24E+01 | 4.28E-05 | SBP |
| rs6062324 | -0.3294 | 0.0363 | 1.18E-19 | 0.7636 | A | G | 757601 | 8.23E+01 | 1.09E-04 | SBP |
| rs6054139 | 0.2094 | 0.0306 | 8.23E-12 | 0.394 | A | G | 757601 | 4.68E+01 | 6.18E-05 | SBP |
| rs2776037 | -0.1851 | 0.0309 | 2.15E-09 | 0.5849 | T | C | 757601 | 3.59E+01 | 4.74E-05 | SBP |
| rs1882961 | 0.2443 | 0.0326 | 6.69E-14 | 0.6913 | T | C | 757601 | 5.62E+01 | 7.41E-05 | SBP |
| rs2833834 | 0.2177 | 0.0338 | 1.22E-10 | 0.7235 | A | C | 757601 | 4.15E+01 | 5.48E-05 | SBP |
| rs12627651 | 0.3498 | 0.0341 | 1.02E-24 | 0.7128 | A | G | 757601 | 1.05E+02 | 1.39E-04 | SBP |
| rs34487963 | -0.8819 | 0.1244 | 1.35E-12 | 0.9815 | A | C | 757601 | 5.03E+01 | 6.63E-05 | SBP |
| rs7278003 | -0.1876 | 0.0304 | 6.63E-10 | 0.5622 | T | C | 757601 | 3.81E+01 | 5.03E-05 | SBP |
| rs2238787 | 0.2552 | 0.0332 | 1.45E-14 | 0.708 | A | G | 757601 | 5.91E+01 | 7.80E-05 | SBP |
| rs113264678 | 0.4063 | 0.0727 | 2.26E-08 | 0.954 | T | C | 757601 | 3.12E+01 | 4.12E-05 | SBP |
| rs8142376 | 0.1676 | 0.03 | 2.20E-08 | 0.509 | T | C | 757601 | 3.12E+01 | 4.12E-05 | SBP |
| rs148140538 | -0.3252 | 0.0562 | 7.39E-09 | 0.9192 | T | C | 757601 | 3.35E+01 | 4.42E-05 | SBP |
| rs28578714 | 0.2066 | 0.0327 | 2.53E-10 | 0.3938 | T | C | 757601 | 3.99E+01 | 5.27E-05 | SBP |
| rs12637573 | -0.1731 | 0.0302 | 9.95E-09 | 0.5282 | A | G | 757601 | 3.29E+01 | 4.34E-05 | SBP |
| rs6438857 | 0.2736 | 0.0305 | 3.13E-19 | 0.4226 | T | C | 757601 | 8.05E+01 | 1.06E-04 | SBP |
| rs9880098 | 0.3081 | 0.0308 | 1.59E-23 | 0.6054 | A | G | 757601 | 1.00E+02 | 1.32E-04 | SBP |
| rs1199330 | -0.2654 | 0.047 | 1.65E-08 | 0.1176 | A | G | 757601 | 3.19E+01 | 4.21E-05 | SBP |
| rs9876694 | 0.4713 | 0.0651 | 4.64E-13 | 0.9416 | T | C | 757601 | 5.24E+01 | 6.92E-05 | SBP |
| rs11925504 | -0.2901 | 0.0305 | 1.78E-21 | 0.4279 | A | G | 757601 | 9.05E+01 | 1.19E-04 | SBP |
| rs4408839 | -0.2301 | 0.0345 | 2.43E-11 | 0.2567 | A | G | 757601 | 4.45E+01 | 5.87E-05 | SBP |
| rs79539362 | 0.4003 | 0.0504 | 2.09E-15 | 0.1008 | T | C | 757601 | 6.31E+01 | 8.33E-05 | SBP |
| rs17684859 | -0.2241 | 0.034 | 4.24E-11 | 0.2665 | T | C | 757601 | 4.34E+01 | 5.73E-05 | SBP |
| rs3980686 | -0.4998 | 0.0487 | 1.03E-24 | 0.8925 | T | G | 757601 | 1.05E+02 | 1.39E-04 | SBP |
| rs1290784 | 0.4124 | 0.0303 | 2.97E-42 | 0.5517 | T | C | 757601 | 1.85E+02 | 2.44E-04 | SBP |
| rs2111557 | 0.1764 | 0.0302 | 5.22E-09 | 0.5325 | T | C | 757601 | 3.41E+01 | 4.50E-05 | SBP |
| rs4955575 | 0.2158 | 0.0348 | 5.63E-10 | 0.2539 | A | C | 757601 | 3.85E+01 | 5.08E-05 | SBP |
| rs262986 | -0.2371 | 0.0305 | 7.67E-15 | 0.5296 | A | G | 757601 | 6.04E+01 | 7.98E-05 | SBP |
| rs9869437 | -0.2001 | 0.0318 | 3.22E-10 | 0.6477 | A | C | 757601 | 3.96E+01 | 5.23E-05 | SBP |
| rs2643826 | 0.4473 | 0.0306 | 1.74E-48 | 0.5495 | T | C | 757601 | 2.14E+02 | 2.82E-04 | SBP |
| rs68115553 | -0.6445 | 0.1143 | 1.74E-08 | 0.0199 | A | G | 757601 | 3.18E+01 | 4.20E-05 | SBP |
| rs743395 | 0.2597 | 0.0317 | 2.55E-16 | 0.6166 | T | C | 757601 | 6.71E+01 | 8.86E-05 | SBP |
| rs6788984 | 0.2999 | 0.0432 | 3.81E-12 | 0.1437 | A | G | 757601 | 4.82E+01 | 6.36E-05 | SBP |
| rs1052501 | 0.2262 | 0.0412 | 4.14E-08 | 0.1671 | T | C | 757601 | 3.01E+01 | 3.98E-05 | SBP |
| rs6771917 | -0.3793 | 0.0355 | 1.39E-26 | 0.7523 | T | C | 757601 | 1.14E+02 | 1.51E-04 | SBP |
| rs7615099 | 0.1891 | 0.0321 | 3.90E-09 | 0.3325 | A | G | 757601 | 3.47E+01 | 4.58E-05 | SBP |
| rs6445583 | 0.2774 | 0.0349 | 1.90E-15 | 0.2535 | A | G | 757601 | 6.32E+01 | 8.34E-05 | SBP |
| rs3772219 | 0.2733 | 0.0324 | 3.10E-17 | 0.3176 | A | C | 757601 | 7.12E+01 | 9.39E-05 | SBP |
| rs1375564 | 0.2579 | 0.0315 | 2.84E-16 | 0.3605 | T | C | 757601 | 6.70E+01 | 8.85E-05 | SBP |
| rs13107325 | -0.9086 | 0.0592 | 4.22E-53 | 0.9261 | T | C | 757601 | 2.36E+02 | 3.11E-04 | SBP |
| rs11097909 | -0.3628 | 0.043 | 3.35E-17 | 0.8528 | T | C | 757601 | 7.12E+01 | 9.40E-05 | SBP |
| rs1493132 | -0.1766 | 0.0318 | 2.73E-08 | 0.3397 | T | C | 757601 | 3.08E+01 | 4.07E-05 | SBP |
| rs1814951 | -0.3231 | 0.0466 | 3.91E-12 | 0.1215 | A | G | 757601 | 4.81E+01 | 6.35E-05 | SBP |
| rs7439567 | 0.2537 | 0.0309 | 2.31E-16 | 0.5894 | T | C | 757601 | 6.74E+01 | 8.90E-05 | SBP |
| rs2353940 | -0.2075 | 0.0358 | 6.85E-09 | 0.2493 | T | C | 757601 | 3.36E+01 | 4.43E-05 | SBP |
| rs73855810 | 0.2732 | 0.0434 | 3.04E-10 | 0.8594 | A | G | 757601 | 3.96E+01 | 5.23E-05 | SBP |
| rs7683728 | -0.3654 | 0.0304 | 2.43E-33 | 0.4688 | T | C | 757601 | 1.44E+02 | 1.91E-04 | SBP |
| rs12643599 | 0.3134 | 0.0313 | 1.23E-23 | 0.3605 | A | G | 757601 | 1.00E+02 | 1.32E-04 | SBP |
| rs17035181 | 0.3074 | 0.0429 | 7.61E-13 | 0.1448 | T | G | 757601 | 5.13E+01 | 6.78E-05 | SBP |
| rs869396 | -0.2115 | 0.0305 | 4.12E-12 | 0.5341 | A | C | 757601 | 4.81E+01 | 6.35E-05 | SBP |
| rs2610990 | -0.2903 | 0.0343 | 2.86E-17 | 0.7359 | A | G | 757601 | 7.16E+01 | 9.45E-05 | SBP |
| rs34535756 | 0.478 | 0.0786 | 1.18E-09 | 0.9606 | T | C | 757601 | 3.70E+01 | 4.88E-05 | SBP |
| rs1290933 | -0.2847 | 0.0327 | 3.17E-18 | 0.3081 | A | C | 757601 | 7.58E+01 | 1.00E-04 | SBP |
| rs55924432 | 0.2651 | 0.0317 | 5.70E-17 | 0.599 | T | C | 757601 | 6.99E+01 | 9.23E-05 | SBP |
| rs2498323 | 0.3171 | 0.0517 | 8.52E-10 | 0.902 | A | G | 757601 | 3.76E+01 | 4.97E-05 | SBP |
| rs2291434 | -0.2622 | 0.0303 | 5.10E-18 | 0.4665 | T | G | 757601 | 7.49E+01 | 9.88E-05 | SBP |
| rs12511987 | -0.2329 | 0.0399 | 5.39E-09 | 0.1774 | T | G | 757601 | 3.41E+01 | 4.50E-05 | SBP |
| rs62309747 | -0.2244 | 0.0304 | 1.59E-13 | 0.5266 | A | G | 757601 | 5.45E+01 | 7.19E-05 | SBP |
| rs60991988 | 0.3789 | 0.0498 | 2.82E-14 | 0.1069 | T | G | 757601 | 5.79E+01 | 7.64E-05 | SBP |
| rs13107261 | -0.1778 | 0.0314 | 1.57E-08 | 0.6313 | A | G | 757601 | 3.21E+01 | 4.23E-05 | SBP |
| rs5020545 | -0.2179 | 0.0305 | 9.71E-13 | 0.5563 | T | C | 757601 | 5.10E+01 | 6.74E-05 | SBP |
| rs12509595 | -0.8367 | 0.0334 | 2.55E-138 | 0.2923 | T | C | 757601 | 6.28E+02 | 8.28E-04 | SBP |
| rs6823199 | 0.2094 | 0.0348 | 1.72E-09 | 0.2562 | T | C | 757601 | 3.62E+01 | 4.78E-05 | SBP |
| rs17010957 | -0.534 | 0.043 | 1.78E-35 | 0.1463 | T | C | 757601 | 1.54E+02 | 2.04E-04 | SBP |
| rs13149209 | 0.281 | 0.0367 | 1.97E-14 | 0.2227 | T | C | 757601 | 5.86E+01 | 7.74E-05 | SBP |
| rs11241313 | -0.2071 | 0.0326 | 2.23E-10 | 0.6888 | T | C | 757601 | 4.04E+01 | 5.33E-05 | SBP |
| rs1624823 | 0.3371 | 0.0313 | 4.26E-27 | 0.6199 | A | G | 757601 | 1.16E+02 | 1.53E-04 | SBP |
| rs6892983 | 0.3427 | 0.0307 | 7.11E-29 | 0.5978 | A | C | 757601 | 1.25E+02 | 1.64E-04 | SBP |
| rs10069690 | 0.3098 | 0.0369 | 4.47E-17 | 0.7418 | T | C | 757601 | 7.05E+01 | 9.30E-05 | SBP |
| rs702395 | 0.2318 | 0.0305 | 3.24E-14 | 0.5631 | T | C | 757601 | 5.78E+01 | 7.62E-05 | SBP |
| rs2913920 | 0.2418 | 0.0359 | 1.62E-11 | 0.235 | T | C | 757601 | 4.54E+01 | 5.99E-05 | SBP |
| rs7725413 | -0.1985 | 0.0359 | 3.07E-08 | 0.2301 | T | C | 757601 | 3.06E+01 | 4.04E-05 | SBP |
| rs1957563 | 0.3629 | 0.0342 | 2.32E-26 | 0.735 | T | C | 757601 | 1.13E+02 | 1.49E-04 | SBP |
| rs11960210 | 0.4727 | 0.0313 | 1.25E-51 | 0.3755 | T | C | 757601 | 2.28E+02 | 3.01E-04 | SBP |
| rs13358657 | -0.388 | 0.0445 | 2.95E-18 | 0.1332 | A | G | 757601 | 7.60E+01 | 1.00E-04 | SBP |
| rs3860770 | -0.2663 | 0.0333 | 1.20E-15 | 0.7084 | A | G | 757601 | 6.40E+01 | 8.44E-05 | SBP |
| rs12153395 | -0.3303 | 0.0486 | 1.07E-11 | 0.8853 | A | G | 757601 | 4.62E+01 | 6.10E-05 | SBP |
| rs12656497 | -0.6382 | 0.0307 | 7.14E-96 | 0.5966 | T | C | 757601 | 4.32E+02 | 5.70E-04 | SBP |
| rs10941043 | -0.2585 | 0.0332 | 6.42E-15 | 0.2902 | T | G | 757601 | 6.06E+01 | 8.00E-05 | SBP |
| rs4957026 | 0.1982 | 0.0323 | 8.12E-10 | 0.6601 | A | G | 757601 | 3.77E+01 | 4.97E-05 | SBP |
| rs2113077 | 0.2097 | 0.0305 | 6.09E-12 | 0.5697 | A | G | 757601 | 4.73E+01 | 6.24E-05 | SBP |
| rs10043077 | -0.1931 | 0.0324 | 2.52E-09 | 0.361 | T | C | 757601 | 3.55E+01 | 4.69E-05 | SBP |
| rs34496659 | 0.4545 | 0.0616 | 1.54E-13 | 0.9298 | A | G | 757601 | 5.44E+01 | 7.19E-05 | SBP |
| rs6870654 | 0.2136 | 0.0347 | 7.58E-10 | 0.2546 | T | C | 757601 | 3.79E+01 | 5.00E-05 | SBP |
| rs4286632 | 0.211 | 0.0343 | 7.64E-10 | 0.2694 | A | G | 757601 | 3.78E+01 | 4.99E-05 | SBP |
| rs7703560 | -0.2246 | 0.0333 | 1.51E-11 | 0.2998 | A | G | 757601 | 4.55E+01 | 6.00E-05 | SBP |
| rs246973 | 0.2479 | 0.0335 | 1.45E-13 | 0.7118 | T | C | 757601 | 5.48E+01 | 7.23E-05 | SBP |
| rs6452769 | -0.3143 | 0.0377 | 7.82E-17 | 0.7947 | A | G | 757601 | 6.95E+01 | 9.17E-05 | SBP |
| rs1871190 | 0.1954 | 0.0324 | 1.66E-09 | 0.6651 | T | G | 757601 | 3.64E+01 | 4.80E-05 | SBP |
| rs9486916 | 0.2657 | 0.0385 | 5.42E-12 | 0.8021 | T | C | 757601 | 4.76E+01 | 6.29E-05 | SBP |
| rs1630736 | -0.1706 | 0.0309 | 3.52E-08 | 0.535 | T | C | 757601 | 3.05E+01 | 4.02E-05 | SBP |
| rs10782230 | 0.2106 | 0.0302 | 2.91E-12 | 0.5155 | A | G | 757601 | 4.86E+01 | 6.42E-05 | SBP |
| rs9401913 | 0.5202 | 0.0305 | 3.66E-65 | 0.5613 | A | G | 757601 | 2.91E+02 | 3.84E-04 | SBP |
| rs9349379 | 0.2664 | 0.0312 | 1.31E-17 | 0.407 | A | G | 757601 | 7.29E+01 | 9.62E-05 | SBP |
| rs2327429 | 0.2 | 0.0338 | 3.16E-09 | 0.2917 | T | C | 757601 | 3.50E+01 | 4.62E-05 | SBP |
| rs8180684 | 0.2134 | 0.0335 | 1.80E-10 | 0.7104 | T | C | 757601 | 4.06E+01 | 5.36E-05 | SBP |
| rs7765526 | 0.201 | 0.0307 | 5.88E-11 | 0.5367 | A | G | 757601 | 4.29E+01 | 5.66E-05 | SBP |
| rs1293969 | -0.1988 | 0.0347 | 1.03E-08 | 0.2516 | T | C | 757601 | 3.28E+01 | 4.33E-05 | SBP |
| rs509833 | 0.329 | 0.044 | 7.08E-14 | 0.8614 | A | G | 757601 | 5.59E+01 | 7.38E-05 | SBP |
| rs2745599 | 0.2164 | 0.0317 | 8.96E-12 | 0.448 | A | G | 757601 | 4.66E+01 | 6.15E-05 | SBP |
| rs12661036 | -0.2104 | 0.0374 | 1.82E-08 | 0.225 | T | C | 757601 | 3.16E+01 | 4.18E-05 | SBP |
| rs7744902 | -0.4088 | 0.0593 | 5.64E-12 | 0.9234 | A | G | 757601 | 4.75E+01 | 6.27E-05 | SBP |
| rs9368222 | 0.2281 | 0.0339 | 1.84E-11 | 0.7312 | A | C | 757601 | 4.53E+01 | 5.98E-05 | SBP |
| rs2655445 | -0.2018 | 0.0312 | 9.58E-11 | 0.3933 | A | G | 757601 | 4.18E+01 | 5.52E-05 | SBP |
| rs79782817 | 0.5324 | 0.0499 | 1.43E-26 | 0.8973 | T | G | 757601 | 1.14E+02 | 1.50E-04 | SBP |
| rs116025100 | 0.5365 | 0.0851 | 2.86E-10 | 0.9617 | A | G | 757601 | 3.97E+01 | 5.25E-05 | SBP |
| rs2815063 | 0.2755 | 0.0458 | 1.76E-09 | 0.8685 | A | C | 757601 | 3.62E+01 | 4.78E-05 | SBP |
| rs7763558 | 0.3363 | 0.0321 | 1.17E-25 | 0.6759 | A | G | 757601 | 1.10E+02 | 1.45E-04 | SBP |
| rs78648104 | -0.4287 | 0.0541 | 2.37E-15 | 0.0925 | T | C | 757601 | 6.28E+01 | 8.29E-05 | SBP |
| rs1575290 | 0.1973 | 0.0301 | 5.59E-11 | 0.5267 | T | C | 757601 | 4.30E+01 | 5.67E-05 | SBP |
| rs1984195 | 0.2409 | 0.0303 | 1.77E-15 | 0.5113 | A | G | 757601 | 6.32E+01 | 8.34E-05 | SBP |
| rs9361836 | 0.2196 | 0.0324 | 1.25E-11 | 0.6828 | T | C | 757601 | 4.59E+01 | 6.06E-05 | SBP |
| rs6921291 | 0.3575 | 0.0385 | 1.58E-20 | 0.8093 | T | C | 757601 | 8.62E+01 | 1.14E-04 | SBP |
| rs2392929 | -0.7507 | 0.0379 | 1.96E-87 | 0.2027 | T | G | 757601 | 3.92E+02 | 5.18E-04 | SBP |
| rs34072724 | -0.2422 | 0.0303 | 1.37E-15 | 0.5111 | A | G | 757601 | 6.39E+01 | 8.43E-05 | SBP |
| rs35680304 | 0.2694 | 0.031 | 3.76E-18 | 0.4071 | T | C | 757601 | 7.55E+01 | 9.97E-05 | SBP |
| rs75672964 | 0.5885 | 0.0839 | 2.35E-12 | 0.9582 | T | C | 757601 | 4.92E+01 | 6.49E-05 | SBP |
| rs73727605 | 0.3616 | 0.0623 | 6.60E-09 | 0.9337 | A | G | 757601 | 3.37E+01 | 4.45E-05 | SBP |
| rs3918226 | 0.664 | 0.0575 | 8.46E-31 | 0.9189 | T | C | 757601 | 1.33E+02 | 1.76E-04 | SBP |
| rs10224210 | -0.3831 | 0.034 | 1.60E-29 | 0.2789 | T | C | 757601 | 1.27E+02 | 1.68E-04 | SBP |
| rs3807925 | -0.1859 | 0.0319 | 5.39E-09 | 0.3504 | A | G | 757601 | 3.40E+01 | 4.48E-05 | SBP |
| rs28688791 | -0.3222 | 0.038 | 2.34E-17 | 0.1982 | T | C | 757601 | 7.19E+01 | 9.49E-05 | SBP |
| rs6959688 | -0.2344 | 0.031 | 4.22E-14 | 0.4019 | A | G | 757601 | 5.72E+01 | 7.55E-05 | SBP |
| rs10282122 | -0.302 | 0.0327 | 2.46E-20 | 0.3316 | T | C | 757601 | 8.53E+01 | 1.13E-04 | SBP |
| rs3735533 | -0.91 | 0.0577 | 5.29E-56 | 0.9257 | T | C | 757601 | 2.49E+02 | 3.28E-04 | SBP |
| rs977184 | -0.184 | 0.0314 | 4.86E-09 | 0.3748 | T | C | 757601 | 3.43E+01 | 4.53E-05 | SBP |
| rs11977526 | -0.3213 | 0.0312 | 6.62E-25 | 0.5991 | A | G | 757601 | 1.06E+02 | 1.40E-04 | SBP |
| rs73049928 | -0.2382 | 0.0392 | 1.20E-09 | 0.1939 | A | G | 757601 | 3.69E+01 | 4.87E-05 | SBP |
| rs12668436 | -0.2151 | 0.035 | 7.88E-10 | 0.2459 | T | C | 757601 | 3.78E+01 | 4.99E-05 | SBP |
| rs848445 | -0.2025 | 0.0339 | 2.28E-09 | 0.7149 | T | C | 757601 | 3.57E+01 | 4.71E-05 | SBP |
| rs42377 | -0.3153 | 0.0331 | 1.69E-21 | 0.6955 | A | G | 757601 | 9.07E+01 | 1.20E-04 | SBP |
| rs79069610 | -0.4005 | 0.0727 | 3.68E-08 | 0.05 | T | C | 757601 | 3.03E+01 | 4.01E-05 | SBP |
| rs35783704 | -0.4619 | 0.0507 | 8.81E-20 | 0.8958 | A | G | 757601 | 8.30E+01 | 1.10E-04 | SBP |
| rs7830607 | -0.206 | 0.0327 | 3.09E-10 | 0.6954 | A | G | 757601 | 3.97E+01 | 5.24E-05 | SBP |
| rs2470004 | -0.3454 | 0.0392 | 1.28E-18 | 0.1825 | T | C | 757601 | 7.76E+01 | 1.02E-04 | SBP |
| rs4598218 | 0.1911 | 0.0313 | 1.00E-09 | 0.3842 | T | C | 757601 | 3.73E+01 | 4.92E-05 | SBP |
| rs7012866 | -0.2325 | 0.0301 | 1.21E-14 | 0.5009 | T | G | 757601 | 5.97E+01 | 7.87E-05 | SBP |
| rs4440615 | -0.2201 | 0.0312 | 1.87E-12 | 0.3679 | A | G | 757601 | 4.98E+01 | 6.57E-05 | SBP |
| rs4961293 | 0.2268 | 0.0303 | 7.35E-14 | 0.5487 | T | C | 757601 | 5.60E+01 | 7.39E-05 | SBP |
| rs10866828 | 0.2476 | 0.0355 | 3.19E-12 | 0.7504 | T | C | 757601 | 4.86E+01 | 6.42E-05 | SBP |
| rs7821832 | 0.4222 | 0.0348 | 6.67E-34 | 0.2553 | T | G | 757601 | 1.47E+02 | 1.94E-04 | SBP |
| rs77375686 | -0.3467 | 0.0485 | 8.38E-13 | 0.1117 | A | G | 757601 | 5.11E+01 | 6.74E-05 | SBP |
| rs1906672 | 0.2966 | 0.0358 | 1.20E-16 | 0.7681 | A | G | 757601 | 6.86E+01 | 9.06E-05 | SBP |
| rs4873492 | 0.3431 | 0.0403 | 1.61E-17 | 0.8276 | T | C | 757601 | 7.25E+01 | 9.57E-05 | SBP |
| rs2354862 | 0.2507 | 0.0317 | 2.42E-15 | 0.3593 | A | C | 757601 | 6.25E+01 | 8.25E-05 | SBP |
| rs13253358 | 0.2127 | 0.033 | 1.13E-10 | 0.7021 | T | C | 757601 | 4.15E+01 | 5.48E-05 | SBP |
| rs2126474 | -0.2601 | 0.0306 | 1.87E-17 | 0.5875 | T | G | 757601 | 7.23E+01 | 9.54E-05 | SBP |
| rs9918879 | -0.2984 | 0.0499 | 2.28E-09 | 0.897 | T | G | 757601 | 3.58E+01 | 4.72E-05 | SBP |
| rs148401029 | -0.4623 | 0.0848 | 4.97E-08 | 0.9648 | A | C | 757601 | 2.97E+01 | 3.92E-05 | SBP |
| rs10091532 | -0.2067 | 0.0305 | 1.33E-11 | 0.5832 | A | C | 757601 | 4.59E+01 | 6.06E-05 | SBP |
| rs843093 | -0.2085 | 0.0338 | 6.95E-10 | 0.2912 | A | G | 757601 | 3.81E+01 | 5.02E-05 | SBP |
| rs10980408 | -0.7606 | 0.0827 | 3.83E-20 | 0.0359 | T | C | 757601 | 8.46E+01 | 1.12E-04 | SBP |
| rs2900568 | -0.1889 | 0.03 | 2.96E-10 | 0.4816 | T | C | 757601 | 3.96E+01 | 5.23E-05 | SBP |
| rs34025993 | 0.223 | 0.0308 | 4.71E-13 | 0.586 | A | G | 757601 | 5.24E+01 | 6.92E-05 | SBP |
| rs7854147 | 0.3056 | 0.0461 | 3.29E-11 | 0.123 | A | G | 757601 | 4.39E+01 | 5.80E-05 | SBP |
| rs13289468 | 0.2488 | 0.0306 | 3.93E-16 | 0.4257 | A | C | 757601 | 6.61E+01 | 8.73E-05 | SBP |
| rs6271 | -0.5547 | 0.0611 | 1.18E-19 | 0.9265 | T | C | 757601 | 8.24E+01 | 1.09E-04 | SBP |
| rs11145807 | 0.2135 | 0.0322 | 3.54E-11 | 0.5943 | A | G | 757601 | 4.40E+01 | 5.80E-05 | SBP |
| rs9886665 | 0.2048 | 0.0343 | 2.47E-09 | 0.7329 | T | C | 757601 | 3.57E+01 | 4.71E-05 | SBP |
| rs4553000 | -0.2035 | 0.03 | 1.09E-11 | 0.4859 | T | C | 757601 | 4.60E+01 | 6.07E-05 | SBP |
| rs76452347 | -0.2974 | 0.0397 | 7.13E-14 | 0.795 | T | C | 757601 | 5.61E+01 | 7.41E-05 | SBP |
| rs927315 | 0.1689 | 0.0303 | 2.44E-08 | 0.5287 | T | C | 757601 | 3.11E+01 | 4.10E-05 | SBP |
| rs60191654 | -0.2382 | 0.0385 | 5.88E-10 | 0.1882 | A | G | 757601 | 3.83E+01 | 5.05E-05 | SBP |
| rs10746963 | -0.2177 | 0.0388 | 2.05E-08 | 0.8166 | A | G | 757601 | 3.15E+01 | 4.16E-05 | SBP |
| rs1332813 | 0.2203 | 0.0314 | 2.32E-12 | 0.6486 | T | C | 757601 | 4.92E+01 | 6.50E-05 | SBP |
| rs2753960 | 0.4466 | 0.0309 | 2.66E-47 | 0.5801 | T | G | 757601 | 2.09E+02 | 2.76E-04 | SBP |
| rs9508495 | -0.3557 | 0.0353 | 6.34E-24 | 0.2435 | T | C | 757601 | 1.02E+02 | 1.34E-04 | SBP |
| rs488834 | -0.1931 | 0.0208 | 1.94E-20 | 0.2359 | T | C | 757601 | 8.62E+01 | 1.14E-04 | DBP |
| rs10776752 | 0.4573 | 0.033 | 1.25E-43 | 0.9195 | T | G | 757601 | 1.92E+02 | 2.53E-04 | DBP |
| rs55857306 | -0.5224 | 0.0235 | 5.05E-109 | 0.8398 | A | G | 757601 | 4.94E+02 | 6.52E-04 | DBP |
| rs1819663 | 0.1147 | 0.0174 | 4.63E-11 | 0.4929 | A | G | 757601 | 4.35E+01 | 5.74E-05 | DBP |
| rs76719272 | -0.1438 | 0.0264 | 4.86E-08 | 0.8685 | T | C | 757601 | 2.97E+01 | 3.92E-05 | DBP |
| rs1889785 | 0.1255 | 0.0174 | 5.61E-13 | 0.5449 | A | G | 757601 | 5.20E+01 | 6.87E-05 | DBP |
| rs7524019 | 0.1036 | 0.0174 | 2.60E-09 | 0.508 | T | C | 757601 | 3.55E+01 | 4.68E-05 | DBP |
| rs12405515 | -0.1698 | 0.0174 | 1.92E-22 | 0.4298 | T | G | 757601 | 9.52E+01 | 1.26E-04 | DBP |
| rs34645159 | -0.133 | 0.0174 | 2.07E-14 | 0.4987 | A | G | 757601 | 5.84E+01 | 7.71E-05 | DBP |
| rs150816167 | -0.2873 | 0.0446 | 1.17E-10 | 0.0451 | T | C | 757601 | 4.15E+01 | 5.48E-05 | DBP |
| rs4651224 | 0.1102 | 0.0175 | 3.39E-10 | 0.5531 | T | C | 757601 | 3.97E+01 | 5.23E-05 | DBP |
| rs882624 | -0.1571 | 0.0185 | 2.33E-17 | 0.6675 | T | C | 757601 | 7.21E+01 | 9.52E-05 | DBP |
| rs1502358 | -0.1127 | 0.0185 | 1.13E-09 | 0.3187 | A | G | 757601 | 3.71E+01 | 4.90E-05 | DBP |
| rs68085857 | 0.191 | 0.0205 | 9.83E-21 | 0.766 | T | C | 757601 | 8.68E+01 | 1.15E-04 | DBP |
| rs12088448 | -0.1544 | 0.0182 | 2.53E-17 | 0.356 | A | C | 757601 | 7.20E+01 | 9.50E-05 | DBP |
| rs602521 | 0.1351 | 0.0195 | 3.97E-12 | 0.7344 | A | G | 757601 | 4.80E+01 | 6.34E-05 | DBP |
| rs1745417 | 0.1708 | 0.0173 | 4.69E-23 | 0.4807 | T | C | 757601 | 9.75E+01 | 1.29E-04 | DBP |
| rs699 | -0.2359 | 0.0177 | 1.30E-40 | 0.407 | A | G | 757601 | 1.78E+02 | 2.34E-04 | DBP |
| rs3943093 | 0.2477 | 0.0184 | 3.95E-41 | 0.6766 | T | C | 757601 | 1.81E+02 | 2.39E-04 | DBP |
| rs6686889 | 0.1918 | 0.0199 | 6.95E-22 | 0.7467 | T | C | 757601 | 9.29E+01 | 1.23E-04 | DBP |
| rs12728150 | -0.2045 | 0.0318 | 1.28E-10 | 0.081 | A | G | 757601 | 4.14E+01 | 5.46E-05 | DBP |
| rs2493296 | 0.2496 | 0.0254 | 7.45E-23 | 0.8581 | T | C | 757601 | 9.66E+01 | 1.27E-04 | DBP |
| rs2146315 | -0.1197 | 0.0205 | 5.03E-09 | 0.7682 | T | C | 757601 | 3.41E+01 | 4.50E-05 | DBP |
| rs4926901 | 0.0984 | 0.018 | 4.82E-08 | 0.6452 | A | G | 757601 | 2.99E+01 | 3.94E-05 | DBP |
| rs4926923 | 0.1918 | 0.0308 | 4.75E-10 | 0.0883 | T | C | 757601 | 3.88E+01 | 5.12E-05 | DBP |
| rs61772592 | -0.1509 | 0.0261 | 7.42E-09 | 0.1257 | A | G | 757601 | 3.34E+01 | 4.41E-05 | DBP |
| rs10493408 | 0.1584 | 0.0255 | 5.09E-10 | 0.8669 | A | C | 757601 | 3.86E+01 | 5.09E-05 | DBP |
| rs34517439 | -0.2514 | 0.0279 | 2.02E-19 | 0.8801 | A | C | 757601 | 8.12E+01 | 1.07E-04 | DBP |
| rs786921 | -0.1145 | 0.0176 | 8.63E-11 | 0.4043 | A | G | 757601 | 4.23E+01 | 5.59E-05 | DBP |
| rs17396055 | -0.115 | 0.0184 | 4.13E-10 | 0.6676 | A | G | 757601 | 3.91E+01 | 5.16E-05 | DBP |
| rs1006545 | 0.3633 | 0.0275 | 7.96E-40 | 0.1125 | T | G | 757601 | 1.75E+02 | 2.30E-04 | DBP |
| rs2273654 | 0.1165 | 0.0175 | 2.75E-11 | 0.4392 | T | C | 757601 | 4.43E+01 | 5.85E-05 | DBP |
| rs2484294 | 0.3165 | 0.0196 | 1.17E-58 | 0.2673 | A | G | 757601 | 2.61E+02 | 3.44E-04 | DBP |
| rs72842207 | -0.2112 | 0.0211 | 1.10E-23 | 0.7851 | T | C | 757601 | 1.00E+02 | 1.32E-04 | DBP |
| rs11592107 | 0.1203 | 0.0187 | 1.23E-10 | 0.6906 | A | G | 757601 | 4.14E+01 | 5.46E-05 | DBP |
| rs10490923 | 0.1533 | 0.0262 | 5.02E-09 | 0.8743 | A | G | 757601 | 3.42E+01 | 4.52E-05 | DBP |
| rs9419374 | 0.1164 | 0.0185 | 3.44E-10 | 0.646 | A | G | 757601 | 3.96E+01 | 5.23E-05 | DBP |
| rs1133400 | -0.1318 | 0.0215 | 8.30E-10 | 0.2148 | A | G | 757601 | 3.76E+01 | 4.96E-05 | DBP |
| rs6602177 | -0.1203 | 0.0207 | 6.52E-09 | 0.2927 | T | C | 757601 | 3.38E+01 | 4.46E-05 | DBP |
| rs1623474 | 0.2234 | 0.0184 | 6.24E-34 | 0.67 | T | C | 757601 | 1.47E+02 | 1.95E-04 | DBP |
| rs1265842 | 0.1113 | 0.0174 | 1.70E-10 | 0.5166 | T | C | 757601 | 4.09E+01 | 5.40E-05 | DBP |
| rs2487926 | 0.0972 | 0.0176 | 3.31E-08 | 0.4295 | A | G | 757601 | 3.05E+01 | 4.03E-05 | DBP |
| rs3006583 | -0.1303 | 0.0222 | 4.66E-09 | 0.1886 | T | C | 757601 | 3.44E+01 | 4.55E-05 | DBP |
| rs11252324 | -0.2339 | 0.0328 | 1.03E-12 | 0.923 | T | G | 757601 | 5.09E+01 | 6.71E-05 | DBP |
| rs4948643 | 0.1591 | 0.0194 | 2.26E-16 | 0.718 | T | C | 757601 | 6.73E+01 | 8.88E-05 | DBP |
| rs34130368 | -0.2027 | 0.0284 | 8.77E-13 | 0.8828 | T | G | 757601 | 5.09E+01 | 6.72E-05 | DBP |
| rs72831343 | 0.4936 | 0.0248 | 4.77E-88 | 0.1419 | T | G | 757601 | 3.96E+02 | 5.23E-04 | DBP |
| rs2236295 | -0.207 | 0.0177 | 1.42E-31 | 0.6008 | T | G | 757601 | 1.37E+02 | 1.80E-04 | DBP |
| rs35506078 | -0.1348 | 0.0183 | 1.54E-13 | 0.3366 | T | C | 757601 | 5.43E+01 | 7.16E-05 | DBP |
| rs12247028 | -0.1396 | 0.0188 | 1.18E-13 | 0.3678 | A | G | 757601 | 5.51E+01 | 7.28E-05 | DBP |
| rs604723 | -0.3848 | 0.0194 | 2.32E-87 | 0.7247 | T | C | 757601 | 3.93E+02 | 5.19E-04 | DBP |
| rs66682451 | 0.1348 | 0.0194 | 3.44E-12 | 0.2747 | A | G | 757601 | 4.83E+01 | 6.37E-05 | DBP |
| rs7106104 | -0.1186 | 0.0193 | 7.72E-10 | 0.281 | T | C | 757601 | 3.78E+01 | 4.98E-05 | DBP |
| rs12790943 | -0.1002 | 0.0175 | 1.14E-08 | 0.5787 | T | C | 757601 | 3.28E+01 | 4.33E-05 | DBP |
| rs12574332 | 0.2072 | 0.0266 | 6.14E-15 | 0.8773 | T | C | 757601 | 6.07E+01 | 8.01E-05 | DBP |
| rs4936099 | 0.1745 | 0.0178 | 1.16E-22 | 0.4011 | A | C | 757601 | 9.61E+01 | 1.27E-04 | DBP |
| rs4756782 | 0.1551 | 0.0234 | 3.52E-11 | 0.835 | A | C | 757601 | 4.39E+01 | 5.80E-05 | DBP |
| rs28570096 | 0.1396 | 0.0188 | 1.15E-13 | 0.6906 | T | C | 757601 | 5.51E+01 | 7.28E-05 | DBP |
| rs10832586 | -0.3083 | 0.0216 | 2.53E-46 | 0.2016 | A | C | 757601 | 2.04E+02 | 2.69E-04 | DBP |
| rs7926335 | 0.1804 | 0.0195 | 2.05E-20 | 0.7301 | T | C | 757601 | 8.56E+01 | 1.13E-04 | DBP |
| rs569550 | -0.2688 | 0.0181 | 1.23E-49 | 0.3954 | T | G | 757601 | 2.21E+02 | 2.91E-04 | DBP |
| rs147081004 | 0.1411 | 0.0257 | 4.09E-08 | 0.1444 | A | C | 757601 | 3.01E+01 | 3.98E-05 | DBP |
| rs10500932 | 0.2784 | 0.0333 | 5.79E-17 | 0.9257 | A | G | 757601 | 6.99E+01 | 9.23E-05 | DBP |
| rs962369 | 0.1684 | 0.0189 | 6.02E-19 | 0.3013 | T | C | 757601 | 7.94E+01 | 1.05E-04 | DBP |
| rs7933758 | -0.1138 | 0.0191 | 2.58E-09 | 0.6953 | T | C | 757601 | 3.55E+01 | 4.69E-05 | DBP |
| rs10838702 | 0.2375 | 0.0178 | 1.27E-40 | 0.6125 | T | G | 757601 | 1.78E+02 | 2.35E-04 | DBP |
| rs11040503 | -0.17 | 0.0236 | 5.65E-13 | 0.8143 | A | C | 757601 | 5.19E+01 | 6.85E-05 | DBP |
| rs751984 | 0.3937 | 0.0275 | 1.38E-46 | 0.1174 | T | C | 757601 | 2.05E+02 | 2.70E-04 | DBP |
| rs35927325 | 0.2221 | 0.0364 | 1.01E-09 | 0.9386 | T | C | 757601 | 3.72E+01 | 4.91E-05 | DBP |
| rs2306363 | -0.2643 | 0.0216 | 1.63E-34 | 0.7952 | T | G | 757601 | 1.50E+02 | 1.98E-04 | DBP |
| rs11228613 | 0.1741 | 0.0212 | 2.10E-16 | 0.2163 | T | G | 757601 | 6.74E+01 | 8.90E-05 | DBP |
| rs504217 | 0.2745 | 0.0335 | 2.51E-16 | 0.9264 | T | C | 757601 | 6.71E+01 | 8.86E-05 | DBP |
| rs7115331 | -0.1266 | 0.0192 | 3.92E-11 | 0.2857 | T | G | 757601 | 4.35E+01 | 5.74E-05 | DBP |
| rs360153 | -0.2198 | 0.0175 | 4.37E-36 | 0.5828 | T | C | 757601 | 1.58E+02 | 2.08E-04 | DBP |
| rs116063464 | 0.2017 | 0.0369 | 4.68E-08 | 0.9399 | A | G | 757601 | 2.99E+01 | 3.94E-05 | DBP |
| rs7137828 | -0.5027 | 0.0176 | 4.80E-180 | 0.4817 | T | C | 757601 | 8.16E+02 | 1.08E-03 | DBP |
| rs6490019 | -0.1778 | 0.0178 | 2.10E-23 | 0.6203 | A | G | 757601 | 9.98E+01 | 1.32E-04 | DBP |
| rs1790123 | 0.1991 | 0.0218 | 6.87E-20 | 0.1968 | T | C | 757601 | 8.34E+01 | 1.10E-04 | DBP |
| rs2271139 | -0.1247 | 0.0192 | 8.23E-11 | 0.714 | A | C | 757601 | 4.22E+01 | 5.57E-05 | DBP |
| rs6487076 | 0.174 | 0.0209 | 8.69E-17 | 0.223 | A | G | 757601 | 6.93E+01 | 9.15E-05 | DBP |
| rs55935819 | 0.1271 | 0.0181 | 1.96E-12 | 0.6364 | A | G | 757601 | 4.93E+01 | 6.51E-05 | DBP |
| rs12229480 | 0.1359 | 0.0193 | 2.11E-12 | 0.2775 | T | C | 757601 | 4.96E+01 | 6.54E-05 | DBP |
| rs1669907 | 0.1158 | 0.0191 | 1.36E-09 | 0.6968 | T | G | 757601 | 3.68E+01 | 4.85E-05 | DBP |
| rs61917655 | 0.2246 | 0.0297 | 3.72E-14 | 0.8989 | T | C | 757601 | 5.72E+01 | 7.55E-05 | DBP |
| rs7967705 | 0.2694 | 0.0178 | 1.54E-51 | 0.6196 | T | C | 757601 | 2.29E+02 | 3.02E-04 | DBP |
| rs7306947 | -0.205 | 0.0342 | 2.14E-09 | 0.072 | T | G | 757601 | 3.59E+01 | 4.74E-05 | DBP |
| rs6580970 | -0.1661 | 0.0191 | 4.03E-18 | 0.7013 | T | C | 757601 | 7.56E+01 | 9.98E-05 | DBP |
| rs6581101 | -0.1261 | 0.0179 | 2.05E-12 | 0.3962 | A | C | 757601 | 4.96E+01 | 6.55E-05 | DBP |
| rs7959649 | 0.1166 | 0.0202 | 8.14E-09 | 0.7576 | T | C | 757601 | 3.33E+01 | 4.40E-05 | DBP |
| rs521033 | -0.1802 | 0.0253 | 1.10E-12 | 0.1364 | A | G | 757601 | 5.07E+01 | 6.70E-05 | DBP |
| rs710698 | 0.1059 | 0.0176 | 1.89E-09 | 0.4135 | A | G | 757601 | 3.62E+01 | 4.78E-05 | DBP |
| rs2681485 | 0.2945 | 0.0176 | 1.31E-62 | 0.4024 | A | G | 757601 | 2.80E+02 | 3.69E-04 | DBP |
| rs11108209 | -0.1901 | 0.03 | 2.40E-10 | 0.0932 | T | C | 757601 | 4.02E+01 | 5.30E-05 | DBP |
| rs7990017 | 0.1039 | 0.0185 | 1.92E-08 | 0.5267 | T | C | 757601 | 3.15E+01 | 4.16E-05 | DBP |
| rs7491960 | -0.1288 | 0.018 | 8.44E-13 | 0.508 | T | C | 757601 | 5.12E+01 | 6.76E-05 | DBP |
| rs7321688 | 0.1507 | 0.0205 | 1.99E-13 | 0.7675 | A | C | 757601 | 5.40E+01 | 7.13E-05 | DBP |
| rs682681 | -0.1454 | 0.0185 | 4.47E-15 | 0.6665 | T | C | 757601 | 6.18E+01 | 8.15E-05 | DBP |
| rs61948065 | -0.1737 | 0.027 | 1.17E-10 | 0.1212 | A | C | 757601 | 4.14E+01 | 5.46E-05 | DBP |
| rs56256111 | 0.1926 | 0.0263 | 2.60E-13 | 0.8558 | A | G | 757601 | 5.36E+01 | 7.08E-05 | DBP |
| rs7992292 | 0.1367 | 0.0231 | 3.19E-09 | 0.176 | A | G | 757601 | 3.50E+01 | 4.62E-05 | DBP |
| rs9526707 | -0.1217 | 0.0186 | 6.59E-11 | 0.6778 | A | G | 757601 | 4.28E+01 | 5.65E-05 | DBP |
| rs9563529 | 0.1222 | 0.0215 | 1.38E-08 | 0.7957 | T | G | 757601 | 3.23E+01 | 4.26E-05 | DBP |
| rs3861113 | 0.2126 | 0.0322 | 3.95E-11 | 0.9175 | A | C | 757601 | 4.36E+01 | 5.75E-05 | DBP |
| rs12866098 | 0.1033 | 0.0186 | 2.73E-08 | 0.6577 | A | G | 757601 | 3.08E+01 | 4.07E-05 | DBP |
| rs1215469 | -0.1383 | 0.0211 | 5.23E-11 | 0.7705 | A | C | 757601 | 4.30E+01 | 5.67E-05 | DBP |
| rs55684003 | 0.122 | 0.0189 | 1.01E-10 | 0.3041 | A | G | 757601 | 4.17E+01 | 5.50E-05 | DBP |
| rs8014182 | -0.1942 | 0.0257 | 3.94E-14 | 0.8681 | T | C | 757601 | 5.71E+01 | 7.54E-05 | DBP |
| rs7350752 | -0.1504 | 0.0268 | 1.97E-08 | 0.8759 | A | G | 757601 | 3.15E+01 | 4.16E-05 | DBP |
| rs17880989 | 0.4014 | 0.0591 | 1.11E-11 | 0.9741 | A | G | 757601 | 4.61E+01 | 6.09E-05 | DBP |
| rs1950500 | 0.1396 | 0.019 | 2.20E-13 | 0.7081 | T | C | 757601 | 5.40E+01 | 7.13E-05 | DBP |
| rs4424827 | -0.0981 | 0.0175 | 2.11E-08 | 0.4331 | T | C | 757601 | 3.14E+01 | 4.15E-05 | DBP |
| rs7155504 | 0.2286 | 0.0317 | 5.16E-13 | 0.0876 | T | C | 757601 | 5.20E+01 | 6.86E-05 | DBP |
| rs72683923 | 0.5325 | 0.0635 | 5.02E-17 | 0.0212 | T | C | 757601 | 7.03E+01 | 9.28E-05 | DBP |
| rs35413927 | -0.1274 | 0.0189 | 1.77E-11 | 0.3049 | A | G | 757601 | 4.54E+01 | 6.00E-05 | DBP |
| rs12148044 | 0.1371 | 0.023 | 2.66E-09 | 0.8266 | A | G | 757601 | 3.55E+01 | 4.69E-05 | DBP |
| rs227426 | 0.1119 | 0.0175 | 1.75E-10 | 0.4381 | T | G | 757601 | 4.09E+01 | 5.40E-05 | DBP |
| rs2239268 | 0.1097 | 0.019 | 7.40E-09 | 0.2995 | A | G | 757601 | 3.33E+01 | 4.40E-05 | DBP |
| rs4903064 | 0.1543 | 0.0206 | 7.84E-14 | 0.2355 | T | C | 757601 | 5.61E+01 | 7.41E-05 | DBP |
| rs10873612 | -0.1096 | 0.0179 | 9.51E-10 | 0.4039 | T | C | 757601 | 3.75E+01 | 4.95E-05 | DBP |
| rs11070245 | -0.1287 | 0.0174 | 1.57E-13 | 0.5321 | T | G | 757601 | 5.47E+01 | 7.22E-05 | DBP |
| rs2925345 | 0.189 | 0.0174 | 1.60E-27 | 0.5324 | T | C | 757601 | 1.18E+02 | 1.56E-04 | DBP |
| rs17678552 | -0.1649 | 0.0182 | 1.33E-19 | 0.3439 | T | C | 757601 | 8.21E+01 | 1.08E-04 | DBP |
| rs7169864 | -0.1132 | 0.0205 | 3.40E-08 | 0.7678 | T | C | 757601 | 3.05E+01 | 4.02E-05 | DBP |
| rs28429256 | 0.1636 | 0.0188 | 2.83E-18 | 0.6656 | A | G | 757601 | 7.57E+01 | 9.99E-05 | DBP |
| rs2469141 | 0.1351 | 0.0238 | 1.39E-08 | 0.1628 | T | C | 757601 | 3.22E+01 | 4.25E-05 | DBP |
| rs3743111 | 0.1517 | 0.0178 | 1.62E-17 | 0.387 | A | G | 757601 | 7.26E+01 | 9.59E-05 | DBP |
| rs11636952 | 0.3997 | 0.0189 | 5.21E-99 | 0.6869 | T | C | 757601 | 4.47E+02 | 5.90E-04 | DBP |
| rs57708073 | 0.1907 | 0.0214 | 4.73E-19 | 0.2608 | A | G | 757601 | 7.94E+01 | 1.05E-04 | DBP |
| rs2627313 | 0.151 | 0.0175 | 5.86E-18 | 0.5543 | T | C | 757601 | 7.45E+01 | 9.83E-05 | DBP |
| rs77032376 | -0.173 | 0.0249 | 3.64E-12 | 0.8521 | T | C | 757601 | 4.83E+01 | 6.37E-05 | DBP |
| rs4932373 | -0.3664 | 0.0189 | 7.71E-84 | 0.3257 | A | C | 757601 | 3.76E+02 | 4.96E-04 | DBP |
| rs3743369 | 0.104 | 0.0179 | 6.82E-09 | 0.3722 | A | G | 757601 | 3.38E+01 | 4.46E-05 | DBP |
| rs12906962 | -0.2378 | 0.0188 | 8.73E-37 | 0.3233 | T | C | 757601 | 1.60E+02 | 2.11E-04 | DBP |
| rs2589218 | -0.1207 | 0.0196 | 6.90E-10 | 0.2698 | T | C | 757601 | 3.79E+01 | 5.01E-05 | DBP |
| rs77924615 | -0.3163 | 0.0224 | 3.72E-45 | 0.8018 | A | G | 757601 | 1.99E+02 | 2.63E-04 | DBP |
| rs12596630 | 0.2606 | 0.0314 | 1.03E-16 | 0.9095 | T | C | 757601 | 6.89E+01 | 9.09E-05 | DBP |
| rs9937801 | 0.1554 | 0.0174 | 4.81E-19 | 0.4308 | T | C | 757601 | 7.98E+01 | 1.05E-04 | DBP |
| rs80095680 | -0.1566 | 0.0198 | 2.81E-15 | 0.2633 | A | G | 757601 | 6.26E+01 | 8.26E-05 | DBP |
| rs917522 | 0.1665 | 0.0273 | 1.04E-09 | 0.115 | T | C | 757601 | 3.72E+01 | 4.91E-05 | DBP |
| rs12446456 | -0.181 | 0.0175 | 3.99E-25 | 0.5727 | T | C | 757601 | 1.07E+02 | 1.41E-04 | DBP |
| rs7192407 | 0.1019 | 0.0174 | 4.53E-09 | 0.528 | T | C | 757601 | 3.43E+01 | 4.53E-05 | DBP |
| rs62030049 | 0.1336 | 0.0209 | 1.55E-10 | 0.2404 | A | G | 757601 | 4.09E+01 | 5.39E-05 | DBP |
| rs9932220 | -0.1591 | 0.021 | 3.76E-14 | 0.7823 | A | G | 757601 | 5.74E+01 | 7.58E-05 | DBP |
| rs12919839 | -0.1098 | 0.0192 | 1.04E-08 | 0.7159 | T | C | 757601 | 3.27E+01 | 4.32E-05 | DBP |
| rs45474499 | 0.3562 | 0.0415 | 8.50E-18 | 0.9527 | T | C | 757601 | 7.37E+01 | 9.72E-05 | DBP |
| rs28544928 | 0.1543 | 0.0199 | 9.13E-15 | 0.2535 | T | G | 757601 | 6.01E+01 | 7.94E-05 | DBP |
| rs12444212 | 0.1291 | 0.0226 | 1.06E-08 | 0.1826 | T | C | 757601 | 3.26E+01 | 4.31E-05 | DBP |
| rs11859505 | -0.1037 | 0.0181 | 9.76E-09 | 0.5805 | A | G | 757601 | 3.28E+01 | 4.33E-05 | DBP |
| rs8046697 | -0.1289 | 0.0179 | 6.10E-13 | 0.5833 | T | C | 757601 | 5.19E+01 | 6.84E-05 | DBP |
| rs12929303 | 0.1572 | 0.0174 | 1.58E-19 | 0.4675 | A | G | 757601 | 8.16E+01 | 1.08E-04 | DBP |
| rs79286081 | -0.1631 | 0.0299 | 4.83E-08 | 0.8979 | A | G | 757601 | 2.98E+01 | 3.93E-05 | DBP |
| rs908951 | -0.1983 | 0.0181 | 7.73E-28 | 0.563 | T | C | 757601 | 1.20E+02 | 1.58E-04 | DBP |
| rs1043809 | 0.159 | 0.0223 | 9.77E-13 | 0.1917 | T | C | 757601 | 5.08E+01 | 6.71E-05 | DBP |
| rs4362428 | -0.1127 | 0.0176 | 1.45E-10 | 0.5913 | A | C | 757601 | 4.10E+01 | 5.41E-05 | DBP |
| rs76954792 | 0.1213 | 0.0208 | 5.06E-09 | 0.7678 | T | C | 757601 | 3.40E+01 | 4.49E-05 | DBP |
| rs28661492 | -0.1359 | 0.0222 | 9.56E-10 | 0.7978 | T | C | 757601 | 3.75E+01 | 4.95E-05 | DBP |
| rs2239917 | 0.1731 | 0.0176 | 9.69E-23 | 0.5748 | T | C | 757601 | 9.67E+01 | 1.28E-04 | DBP |
| rs3785837 | 0.1453 | 0.0213 | 9.57E-12 | 0.2365 | A | G | 757601 | 4.65E+01 | 6.14E-05 | DBP |
| rs6504163 | -0.1842 | 0.0183 | 6.30E-24 | 0.3763 | T | C | 757601 | 1.01E+02 | 1.34E-04 | DBP |
| rs1867624 | 0.1412 | 0.0178 | 2.08E-15 | 0.3853 | T | C | 757601 | 6.29E+01 | 8.31E-05 | DBP |
| rs12601936 | -0.1429 | 0.0178 | 1.07E-15 | 0.6107 | A | G | 757601 | 6.45E+01 | 8.51E-05 | DBP |
| rs1436138 | 0.1991 | 0.0182 | 7.33E-28 | 0.3633 | A | G | 757601 | 1.20E+02 | 1.58E-04 | DBP |
| rs7217916 | 0.1111 | 0.0179 | 5.63E-10 | 0.6146 | A | G | 757601 | 3.85E+01 | 5.08E-05 | DBP |
| rs138420351 | 0.5568 | 0.0854 | 7.11E-11 | 0.984 | T | C | 757601 | 4.25E+01 | 5.61E-05 | DBP |
| rs74439044 | -0.3496 | 0.0294 | 1.38E-32 | 0.0983 | T | C | 757601 | 1.41E+02 | 1.87E-04 | DBP |
| rs11077961 | 0.1073 | 0.0186 | 8.55E-09 | 0.3676 | A | G | 757601 | 3.33E+01 | 4.39E-05 | DBP |
| rs10164193 | -0.2196 | 0.0327 | 1.87E-11 | 0.0777 | T | G | 757601 | 4.51E+01 | 5.95E-05 | DBP |
| rs11661473 | 0.2007 | 0.0196 | 1.54E-24 | 0.7317 | A | G | 757601 | 1.05E+02 | 1.38E-04 | DBP |
| rs58693787 | 0.1584 | 0.0202 | 3.82E-15 | 0.2458 | A | G | 757601 | 6.15E+01 | 8.12E-05 | DBP |
| rs4102481 | -0.1248 | 0.019 | 4.87E-11 | 0.3049 | T | G | 757601 | 4.31E+01 | 5.69E-05 | DBP |
| rs1903752 | -0.0987 | 0.0178 | 3.20E-08 | 0.4614 | T | C | 757601 | 3.07E+01 | 4.06E-05 | DBP |
| rs4891258 | -0.1159 | 0.0187 | 5.72E-10 | 0.3174 | A | G | 757601 | 3.84E+01 | 5.07E-05 | DBP |
| rs7227492 | 0.181 | 0.0227 | 1.43E-15 | 0.1822 | T | C | 757601 | 6.36E+01 | 8.39E-05 | DBP |
| rs387865 | -0.1059 | 0.0191 | 3.17E-08 | 0.6938 | T | C | 757601 | 3.07E+01 | 4.06E-05 | DBP |
| rs167479 | -0.362 | 0.0188 | 1.67E-82 | 0.5278 | T | G | 757601 | 3.71E+02 | 4.89E-04 | DBP |
| rs1077795 | 0.1987 | 0.0199 | 1.62E-23 | 0.2611 | A | G | 757601 | 9.97E+01 | 1.32E-04 | DBP |
| rs72999033 | 0.2793 | 0.0358 | 5.95E-15 | 0.9342 | T | C | 757601 | 6.09E+01 | 8.03E-05 | DBP |
| rs7257694 | 0.1837 | 0.0178 | 6.28E-25 | 0.5997 | T | C | 757601 | 1.07E+02 | 1.41E-04 | DBP |
| rs8108717 | 0.1323 | 0.0179 | 1.39E-13 | 0.6084 | A | G | 757601 | 5.46E+01 | 7.21E-05 | DBP |
| rs2548459 | -0.132 | 0.0176 | 5.95E-14 | 0.5195 | T | C | 757601 | 5.63E+01 | 7.42E-05 | DBP |
| rs73046792 | -0.1518 | 0.0245 | 5.87E-10 | 0.8408 | A | G | 757601 | 3.84E+01 | 5.07E-05 | DBP |
| rs10424224 | 0.1042 | 0.0182 | 1.05E-08 | 0.6416 | T | C | 757601 | 3.28E+01 | 4.33E-05 | DBP |
| rs7258382 | 0.2624 | 0.0248 | 3.03E-26 | 0.1611 | T | C | 757601 | 1.12E+02 | 1.48E-04 | DBP |
| rs2009733 | 0.1217 | 0.0176 | 5.10E-12 | 0.5005 | A | G | 757601 | 4.78E+01 | 6.31E-05 | DBP |
| rs28377357 | -0.1243 | 0.019 | 6.03E-11 | 0.7062 | A | G | 757601 | 4.28E+01 | 5.65E-05 | DBP |
| rs62158170 | 0.1645 | 0.0211 | 6.63E-15 | 0.2166 | A | G | 757601 | 6.08E+01 | 8.02E-05 | DBP |
| rs13001283 | 0.1522 | 0.0239 | 1.92E-10 | 0.8404 | A | G | 757601 | 4.06E+01 | 5.35E-05 | DBP |
| rs4954192 | -0.1225 | 0.0179 | 8.15E-12 | 0.6128 | T | C | 757601 | 4.68E+01 | 6.18E-05 | DBP |
| rs55944332 | -0.2365 | 0.0204 | 3.27E-31 | 0.2368 | A | G | 757601 | 1.34E+02 | 1.77E-04 | DBP |
| rs12990959 | -0.1271 | 0.0187 | 1.11E-11 | 0.3125 | T | C | 757601 | 4.62E+01 | 6.10E-05 | DBP |
| rs2444769 | 0.158 | 0.0219 | 4.85E-13 | 0.2051 | A | C | 757601 | 5.21E+01 | 6.87E-05 | DBP |
| rs7572130 | -0.1796 | 0.0287 | 4.12E-10 | 0.1042 | A | G | 757601 | 3.92E+01 | 5.17E-05 | DBP |
| rs75717699 | -0.4667 | 0.0541 | 6.71E-18 | 0.0305 | T | G | 757601 | 7.44E+01 | 9.82E-05 | DBP |
| rs1518460 | 0.1342 | 0.0189 | 1.26E-12 | 0.2918 | A | G | 757601 | 5.04E+01 | 6.65E-05 | DBP |
| rs12693302 | -0.2378 | 0.0181 | 2.16E-39 | 0.3482 | A | G | 757601 | 1.73E+02 | 2.28E-04 | DBP |
| rs7592578 | -0.1998 | 0.0224 | 4.71E-19 | 0.8062 | T | G | 757601 | 7.96E+01 | 1.05E-04 | DBP |
| rs824523 | 0.1226 | 0.0183 | 2.26E-11 | 0.6656 | A | C | 757601 | 4.49E+01 | 5.92E-05 | DBP |
| rs11692619 | -0.1281 | 0.0184 | 3.31E-12 | 0.6393 | T | C | 757601 | 4.85E+01 | 6.40E-05 | DBP |
| rs1263671 | -0.1394 | 0.0238 | 4.69E-09 | 0.1632 | T | C | 757601 | 3.43E+01 | 4.53E-05 | DBP |
| rs4675682 | -0.1409 | 0.0173 | 4.49E-16 | 0.4622 | T | C | 757601 | 6.63E+01 | 8.75E-05 | DBP |
| rs1035673 | 0.1625 | 0.0176 | 3.00E-20 | 0.6032 | T | C | 757601 | 8.52E+01 | 1.13E-04 | DBP |
| rs1039897 | -0.1085 | 0.0183 | 3.26E-09 | 0.3497 | A | G | 757601 | 3.52E+01 | 4.64E-05 | DBP |
| rs10804330 | 0.1331 | 0.0176 | 4.60E-14 | 0.4329 | T | C | 757601 | 5.72E+01 | 7.55E-05 | DBP |
| rs1044822 | -0.1334 | 0.0243 | 4.14E-08 | 0.8512 | T | C | 757601 | 3.01E+01 | 3.98E-05 | DBP |
| rs4507125 | -0.1244 | 0.0211 | 3.60E-09 | 0.2136 | A | C | 757601 | 3.48E+01 | 4.59E-05 | DBP |
| rs11687089 | 0.1739 | 0.0175 | 2.79E-23 | 0.4171 | T | C | 757601 | 9.87E+01 | 1.30E-04 | DBP |
| rs1275988 | -0.2945 | 0.0177 | 1.92E-62 | 0.3889 | T | C | 757601 | 2.77E+02 | 3.65E-04 | DBP |
| rs11684340 | 0.1249 | 0.021 | 2.75E-09 | 0.2176 | A | C | 757601 | 3.54E+01 | 4.67E-05 | DBP |
| rs76326501 | 0.3618 | 0.0305 | 2.17E-32 | 0.0911 | A | C | 757601 | 1.41E+02 | 1.86E-04 | DBP |
| rs4952668 | -0.192 | 0.018 | 1.13E-26 | 0.3763 | A | G | 757601 | 1.14E+02 | 1.50E-04 | DBP |
| rs2586970 | -0.1493 | 0.0175 | 1.56E-17 | 0.5639 | A | G | 757601 | 7.28E+01 | 9.61E-05 | DBP |
| rs2421200 | -0.1097 | 0.0173 | 2.59E-10 | 0.5118 | T | G | 757601 | 4.02E+01 | 5.31E-05 | DBP |
| rs1876490 | 0.1364 | 0.0192 | 1.16E-12 | 0.2833 | A | G | 757601 | 5.05E+01 | 6.66E-05 | DBP |
| rs6546810 | -0.12 | 0.0181 | 3.16E-11 | 0.3525 | T | C | 757601 | 4.40E+01 | 5.80E-05 | DBP |
| rs311564 | -0.133 | 0.0183 | 4.23E-13 | 0.6539 | A | G | 757601 | 5.28E+01 | 6.97E-05 | DBP |
| rs62155750 | -0.2177 | 0.0196 | 8.27E-29 | 0.3074 | A | G | 757601 | 1.23E+02 | 1.63E-04 | DBP |
| rs693974 | -0.1847 | 0.0177 | 1.76E-25 | 0.3964 | T | C | 757601 | 1.09E+02 | 1.44E-04 | DBP |
| rs1327235 | -0.3018 | 0.0173 | 4.76E-68 | 0.4714 | A | G | 757601 | 3.04E+02 | 4.02E-04 | DBP |
| rs6078393 | 0.1205 | 0.0176 | 7.66E-12 | 0.4106 | T | G | 757601 | 4.69E+01 | 6.19E-05 | DBP |
| rs4814837 | -0.1003 | 0.0184 | 4.62E-08 | 0.6576 | T | C | 757601 | 2.97E+01 | 3.92E-05 | DBP |
| rs2376997 | -0.1389 | 0.0218 | 1.94E-10 | 0.7511 | A | C | 757601 | 4.06E+01 | 5.36E-05 | DBP |
| rs13042148 | -0.1674 | 0.0244 | 7.24E-12 | 0.8463 | T | C | 757601 | 4.71E+01 | 6.21E-05 | DBP |
| rs7265695 | 0.1967 | 0.0219 | 2.48E-19 | 0.1965 | T | C | 757601 | 8.07E+01 | 1.06E-04 | DBP |
| rs6031431 | -0.1153 | 0.0175 | 4.94E-11 | 0.4622 | A | G | 757601 | 4.34E+01 | 5.73E-05 | DBP |
| rs2598 | 0.1387 | 0.0175 | 1.94E-15 | 0.4674 | A | G | 757601 | 6.28E+01 | 8.29E-05 | DBP |
| rs234623 | -0.1191 | 0.0174 | 8.56E-12 | 0.4959 | A | G | 757601 | 4.69E+01 | 6.18E-05 | DBP |
| rs79208229 | 0.2128 | 0.0326 | 6.53E-11 | 0.9125 | T | G | 757601 | 4.26E+01 | 5.62E-05 | DBP |
| rs35213536 | 0.2044 | 0.0205 | 2.54E-23 | 0.7533 | T | G | 757601 | 9.94E+01 | 1.31E-04 | DBP |
| rs6108168 | -0.1901 | 0.0199 | 1.10E-21 | 0.7454 | A | C | 757601 | 9.13E+01 | 1.20E-04 | DBP |
| rs1882961 | 0.1272 | 0.0188 | 1.40E-11 | 0.6912 | T | C | 757601 | 4.58E+01 | 6.04E-05 | DBP |
| rs34487963 | -0.5734 | 0.0712 | 8.18E-16 | 0.9815 | A | C | 757601 | 6.49E+01 | 8.56E-05 | DBP |
| rs7278003 | -0.1293 | 0.0176 | 1.78E-13 | 0.5615 | T | C | 757601 | 5.40E+01 | 7.12E-05 | DBP |
| rs5992929 | 0.1684 | 0.0193 | 3.07E-18 | 0.7166 | T | C | 757601 | 7.61E+01 | 1.00E-04 | DBP |
| rs134041 | 0.1223 | 0.0175 | 3.05E-12 | 0.564 | T | C | 757601 | 4.88E+01 | 6.45E-05 | DBP |
| rs5753630 | 0.107 | 0.0175 | 8.76E-10 | 0.4382 | A | G | 757601 | 3.74E+01 | 4.93E-05 | DBP |
| rs28675079 | -0.1444 | 0.0222 | 8.34E-11 | 0.8133 | A | G | 757601 | 4.23E+01 | 5.58E-05 | DBP |
| rs347585 | 0.1506 | 0.0189 | 1.57E-15 | 0.2986 | T | C | 757601 | 6.35E+01 | 8.38E-05 | DBP |
| rs12152463 | 0.1006 | 0.0174 | 8.02E-09 | 0.5749 | T | C | 757601 | 3.34E+01 | 4.41E-05 | DBP |
| rs4141663 | -0.1496 | 0.0175 | 1.41E-17 | 0.5784 | T | C | 757601 | 7.31E+01 | 9.65E-05 | DBP |
| rs4077158 | -0.1832 | 0.0173 | 3.09E-26 | 0.5286 | T | C | 757601 | 1.12E+02 | 1.48E-04 | DBP |
| rs9289557 | -0.119 | 0.0207 | 8.68E-09 | 0.7396 | T | C | 757601 | 3.30E+01 | 4.36E-05 | DBP |
| rs6763931 | 0.1383 | 0.0173 | 1.48E-15 | 0.5562 | A | G | 757601 | 6.39E+01 | 8.43E-05 | DBP |
| rs1687295 | 0.2061 | 0.0194 | 2.99E-26 | 0.7296 | T | C | 757601 | 1.13E+02 | 1.49E-04 | DBP |
| rs36117336 | -0.147 | 0.0198 | 1.10E-13 | 0.2562 | T | C | 757601 | 5.51E+01 | 7.28E-05 | DBP |
| rs78809139 | -0.2281 | 0.0288 | 2.58E-15 | 0.8986 | A | G | 757601 | 6.27E+01 | 8.28E-05 | DBP |
| rs78151625 | -0.1869 | 0.0233 | 1.04E-15 | 0.1658 | T | C | 757601 | 6.43E+01 | 8.49E-05 | DBP |
| rs62234672 | 0.1248 | 0.0229 | 4.92E-08 | 0.8248 | A | C | 757601 | 2.97E+01 | 3.92E-05 | DBP |
| rs16853198 | 0.3386 | 0.0327 | 4.44E-25 | 0.0762 | A | G | 757601 | 1.07E+02 | 1.42E-04 | DBP |
| rs62294352 | -0.1605 | 0.0223 | 6.07E-13 | 0.7843 | T | C | 757601 | 5.18E+01 | 6.84E-05 | DBP |
| rs6779368 | -0.1791 | 0.0184 | 2.28E-22 | 0.3423 | A | G | 757601 | 9.47E+01 | 1.25E-04 | DBP |
| rs6777317 | 0.1249 | 0.0195 | 1.51E-10 | 0.7101 | A | G | 757601 | 4.10E+01 | 5.41E-05 | DBP |
| rs2643826 | 0.1857 | 0.0175 | 2.83E-26 | 0.5492 | T | C | 757601 | 1.13E+02 | 1.49E-04 | DBP |
| rs7427249 | -0.1098 | 0.0176 | 4.34E-10 | 0.42 | A | G | 757601 | 3.89E+01 | 5.14E-05 | DBP |
| rs3864004 | 0.1004 | 0.0173 | 6.28E-09 | 0.5315 | A | G | 757601 | 3.37E+01 | 4.45E-05 | DBP |
| rs6442105 | -0.2485 | 0.0185 | 3.10E-41 | 0.6726 | A | G | 757601 | 1.80E+02 | 2.38E-04 | DBP |
| rs6445590 | 0.1284 | 0.0174 | 1.65E-13 | 0.5455 | A | G | 757601 | 5.45E+01 | 7.19E-05 | DBP |
| rs3772219 | 0.1754 | 0.0185 | 2.94E-21 | 0.3193 | A | C | 757601 | 8.99E+01 | 1.19E-04 | DBP |
| rs1675383 | 0.1488 | 0.0174 | 1.47E-17 | 0.5569 | A | C | 757601 | 7.31E+01 | 9.65E-05 | DBP |
| rs3774702 | 0.147 | 0.0228 | 1.18E-10 | 0.8232 | A | G | 757601 | 4.16E+01 | 5.49E-05 | DBP |
| rs6795735 | -0.1438 | 0.0176 | 3.05E-16 | 0.5891 | T | C | 757601 | 6.68E+01 | 8.81E-05 | DBP |
| rs7623706 | 0.0975 | 0.0176 | 2.84E-08 | 0.4349 | A | G | 757601 | 3.07E+01 | 4.05E-05 | DBP |
| rs11923343 | -0.1138 | 0.0181 | 3.10E-10 | 0.6396 | A | G | 757601 | 3.95E+01 | 5.22E-05 | DBP |
| rs13107325 | -0.6747 | 0.0339 | 3.72E-88 | 0.9258 | T | C | 757601 | 3.96E+02 | 5.23E-04 | DBP |
| rs12503341 | -0.2993 | 0.0462 | 9.43E-11 | 0.9606 | A | G | 757601 | 4.20E+01 | 5.54E-05 | DBP |
| rs13118687 | -0.1496 | 0.0175 | 1.37E-17 | 0.5298 | A | G | 757601 | 7.31E+01 | 9.65E-05 | DBP |
| rs66887589 | -0.161 | 0.0174 | 1.83E-20 | 0.4779 | T | C | 757601 | 8.56E+01 | 1.13E-04 | DBP |
| rs9286351 | -0.1412 | 0.0177 | 1.61E-15 | 0.4188 | A | G | 757601 | 6.36E+01 | 8.40E-05 | DBP |
| rs72719149 | -0.1279 | 0.0186 | 6.34E-12 | 0.3164 | T | C | 757601 | 4.73E+01 | 6.24E-05 | DBP |
| rs13124515 | -0.1052 | 0.0187 | 1.98E-08 | 0.6869 | T | C | 757601 | 3.16E+01 | 4.18E-05 | DBP |
| rs13139571 | -0.2408 | 0.0203 | 2.29E-32 | 0.7634 | A | C | 757601 | 1.41E+02 | 1.86E-04 | DBP |
| rs1425486 | -0.1331 | 0.0187 | 1.11E-12 | 0.6793 | T | C | 757601 | 5.07E+01 | 6.69E-05 | DBP |
| rs61789369 | -0.3039 | 0.0436 | 3.07E-12 | 0.0435 | A | G | 757601 | 4.86E+01 | 6.41E-05 | DBP |
| rs11721984 | -0.1409 | 0.0177 | 1.89E-15 | 0.5468 | T | C | 757601 | 6.34E+01 | 8.36E-05 | DBP |
| rs62301873 | -0.1734 | 0.0284 | 1.06E-09 | 0.1061 | A | G | 757601 | 3.73E+01 | 4.92E-05 | DBP |
| rs11945489 | -0.1392 | 0.0192 | 3.99E-13 | 0.7091 | T | C | 757601 | 5.26E+01 | 6.94E-05 | DBP |
| rs13152154 | -0.1186 | 0.0195 | 1.23E-09 | 0.2707 | T | C | 757601 | 3.70E+01 | 4.88E-05 | DBP |
| rs12509595 | -0.4972 | 0.0192 | 1.58E-148 | 0.2924 | T | C | 757601 | 6.71E+02 | 8.84E-04 | DBP |
| rs72976750 | -0.1718 | 0.0251 | 7.37E-12 | 0.1396 | T | C | 757601 | 4.68E+01 | 6.18E-05 | DBP |
| rs9326869 | 0.1096 | 0.02 | 3.99E-08 | 0.7513 | T | C | 757601 | 3.00E+01 | 3.96E-05 | DBP |
| rs1582931 | 0.2161 | 0.0175 | 4.51E-35 | 0.5252 | A | G | 757601 | 1.52E+02 | 2.01E-04 | DBP |
| rs17677603 | -0.2 | 0.0178 | 3.90E-29 | 0.3837 | A | G | 757601 | 1.26E+02 | 1.67E-04 | DBP |
| rs10069690 | 0.1615 | 0.021 | 1.42E-14 | 0.7419 | T | C | 757601 | 5.91E+01 | 7.81E-05 | DBP |
| rs55747751 | -0.2239 | 0.0331 | 1.39E-11 | 0.919 | A | G | 757601 | 4.58E+01 | 6.04E-05 | DBP |
| rs4912840 | -0.1485 | 0.0245 | 1.25E-09 | 0.8453 | A | G | 757601 | 3.67E+01 | 4.85E-05 | DBP |
| rs3776299 | 0.1266 | 0.0175 | 5.06E-13 | 0.5441 | A | G | 757601 | 5.23E+01 | 6.91E-05 | DBP |
| rs78909293 | 0.321 | 0.0429 | 7.31E-14 | 0.0449 | T | C | 757601 | 5.60E+01 | 7.39E-05 | DBP |
| rs2921604 | -0.096 | 0.0176 | 4.46E-08 | 0.4633 | T | C | 757601 | 2.98E+01 | 3.93E-05 | DBP |
| rs3117736 | 0.2374 | 0.0196 | 9.71E-34 | 0.7339 | T | C | 757601 | 1.47E+02 | 1.94E-04 | DBP |
| rs11960210 | 0.2474 | 0.018 | 3.36E-43 | 0.3751 | T | C | 757601 | 1.89E+02 | 2.49E-04 | DBP |
| rs13358657 | -0.224 | 0.0255 | 1.70E-18 | 0.1332 | A | G | 757601 | 7.72E+01 | 1.02E-04 | DBP |
| rs6556384 | -0.152 | 0.0221 | 5.91E-12 | 0.1895 | A | C | 757601 | 4.73E+01 | 6.24E-05 | DBP |
| rs114503346 | -0.2678 | 0.0426 | 3.10E-10 | 0.9539 | T | C | 757601 | 3.95E+01 | 5.22E-05 | DBP |
| rs55993676 | -0.2097 | 0.0191 | 3.82E-28 | 0.7084 | T | G | 757601 | 1.21E+02 | 1.59E-04 | DBP |
| rs10941043 | -0.1269 | 0.019 | 2.52E-11 | 0.2906 | T | G | 757601 | 4.46E+01 | 5.89E-05 | DBP |
| rs4645335 | 0.1142 | 0.0185 | 7.04E-10 | 0.664 | A | G | 757601 | 3.81E+01 | 5.03E-05 | DBP |
| rs7737851 | -0.1256 | 0.022 | 1.11E-08 | 0.8056 | T | C | 757601 | 3.26E+01 | 4.30E-05 | DBP |
| rs6875967 | 0.1344 | 0.0181 | 1.21E-13 | 0.6479 | A | G | 757601 | 5.51E+01 | 7.28E-05 | DBP |
| rs10054208 | 0.1187 | 0.0185 | 1.49E-10 | 0.6383 | T | C | 757601 | 4.12E+01 | 5.43E-05 | DBP |
| rs12515541 | 0.1156 | 0.0177 | 6.23E-11 | 0.3928 | T | G | 757601 | 4.27E+01 | 5.63E-05 | DBP |
| rs1848510 | 0.1256 | 0.0181 | 4.10E-12 | 0.6377 | A | G | 757601 | 4.82E+01 | 6.36E-05 | DBP |
| rs10062049 | 0.2208 | 0.0255 | 4.50E-18 | 0.8641 | T | C | 757601 | 7.50E+01 | 9.90E-05 | DBP |
| rs2307111 | -0.1742 | 0.0178 | 1.62E-22 | 0.3966 | T | C | 757601 | 9.58E+01 | 1.26E-04 | DBP |
| rs4704514 | 0.1087 | 0.0193 | 1.71E-08 | 0.7167 | T | C | 757601 | 3.17E+01 | 4.19E-05 | DBP |
| rs62380354 | 0.1825 | 0.0291 | 3.68E-10 | 0.1096 | A | C | 757601 | 3.93E+01 | 5.19E-05 | DBP |
| rs13355146 | 0.1224 | 0.0178 | 6.39E-12 | 0.6168 | T | C | 757601 | 4.73E+01 | 6.24E-05 | DBP |
| rs55770741 | -0.1281 | 0.0175 | 2.20E-13 | 0.4387 | T | C | 757601 | 5.36E+01 | 7.07E-05 | DBP |
| rs1871190 | 0.1078 | 0.0186 | 6.63E-09 | 0.6656 | T | G | 757601 | 3.36E+01 | 4.43E-05 | DBP |
| rs7767235 | -0.1181 | 0.0182 | 7.95E-11 | 0.6468 | A | C | 757601 | 4.21E+01 | 5.56E-05 | DBP |
| rs509067 | -0.1436 | 0.0175 | 2.65E-16 | 0.5863 | T | C | 757601 | 6.73E+01 | 8.89E-05 | DBP |
| rs11153730 | 0.1551 | 0.0173 | 2.57E-19 | 0.4906 | T | C | 757601 | 8.04E+01 | 1.06E-04 | DBP |
| rs76785130 | -0.4285 | 0.0662 | 9.36E-11 | 0.0199 | A | G | 757601 | 4.19E+01 | 5.53E-05 | DBP |
| rs13215166 | -0.3094 | 0.0174 | 1.79E-70 | 0.4415 | A | G | 757601 | 3.16E+02 | 4.17E-04 | DBP |
| rs9399137 | 0.1148 | 0.0197 | 5.83E-09 | 0.2619 | T | C | 757601 | 3.40E+01 | 4.48E-05 | DBP |
| rs636202 | 0.1023 | 0.0174 | 4.40E-09 | 0.5185 | T | C | 757601 | 3.46E+01 | 4.56E-05 | DBP |
| rs9791312 | -0.1225 | 0.0184 | 2.89E-11 | 0.3452 | A | C | 757601 | 4.43E+01 | 5.85E-05 | DBP |
| rs62434124 | -0.4853 | 0.0338 | 7.83E-47 | 0.9289 | T | C | 757601 | 2.06E+02 | 2.72E-04 | DBP |
| rs9478282 | -0.1994 | 0.0279 | 8.70E-13 | 0.8884 | T | C | 757601 | 5.11E+01 | 6.74E-05 | DBP |
| rs2569882 | 0.1199 | 0.0182 | 4.28E-11 | 0.4342 | T | C | 757601 | 4.34E+01 | 5.73E-05 | DBP |
| rs9365555 | 0.1254 | 0.0187 | 1.96E-11 | 0.3259 | A | G | 757601 | 4.50E+01 | 5.94E-05 | DBP |
| rs11961593 | -0.3158 | 0.0349 | 1.49E-19 | 0.9315 | T | C | 757601 | 8.19E+01 | 1.08E-04 | DBP |
| rs1322639 | -0.1584 | 0.0209 | 3.87E-14 | 0.2234 | A | G | 757601 | 5.74E+01 | 7.58E-05 | DBP |
| rs35261542 | 0.1196 | 0.0195 | 9.29E-10 | 0.7321 | A | C | 757601 | 3.76E+01 | 4.97E-05 | DBP |
| rs6934891 | 0.1275 | 0.0177 | 5.21E-13 | 0.5745 | A | G | 757601 | 5.19E+01 | 6.85E-05 | DBP |
| rs2744133 | 0.1435 | 0.0193 | 1.17E-13 | 0.2749 | A | G | 757601 | 5.53E+01 | 7.30E-05 | DBP |
| rs198851 | 0.3889 | 0.0244 | 2.93E-57 | 0.8504 | T | G | 757601 | 2.54E+02 | 3.35E-04 | DBP |
| rs115447786 | 0.2904 | 0.0455 | 1.75E-10 | 0.9573 | T | C | 757601 | 4.07E+01 | 5.38E-05 | DBP |
| rs6905288 | 0.1759 | 0.0179 | 7.79E-23 | 0.4319 | A | G | 757601 | 9.66E+01 | 1.27E-04 | DBP |
| rs881858 | 0.1553 | 0.0191 | 4.65E-16 | 0.306 | A | G | 757601 | 6.61E+01 | 8.73E-05 | DBP |
| rs2397060 | -0.161 | 0.0251 | 1.46E-10 | 0.1405 | T | C | 757601 | 4.11E+01 | 5.43E-05 | DBP |
| rs1114347 | -0.1792 | 0.0173 | 3.32E-25 | 0.4823 | A | G | 757601 | 1.07E+02 | 1.42E-04 | DBP |
| rs62413546 | -0.1877 | 0.032 | 4.58E-09 | 0.9153 | T | C | 757601 | 3.44E+01 | 4.54E-05 | DBP |
| rs504691 | -0.1177 | 0.0177 | 3.14E-11 | 0.5998 | A | C | 757601 | 4.42E+01 | 5.84E-05 | DBP |
| rs1984195 | 0.1736 | 0.0173 | 1.43E-23 | 0.5117 | A | G | 757601 | 1.01E+02 | 1.33E-04 | DBP |
| rs9406076 | 0.101 | 0.0185 | 4.65E-08 | 0.6722 | T | C | 757601 | 2.98E+01 | 3.93E-05 | DBP |
| rs16875357 | -0.1205 | 0.0203 | 2.70E-09 | 0.2431 | T | G | 757601 | 3.52E+01 | 4.65E-05 | DBP |
| rs3798293 | -0.1328 | 0.021 | 2.70E-10 | 0.2165 | A | G | 757601 | 4.00E+01 | 5.28E-05 | DBP |
| rs4556017 | -0.1601 | 0.0247 | 9.67E-11 | 0.1476 | T | C | 757601 | 4.20E+01 | 5.55E-05 | DBP |
| rs2191046 | 0.1184 | 0.0197 | 1.78E-09 | 0.2646 | T | G | 757601 | 3.61E+01 | 4.77E-05 | DBP |
| rs11556924 | -0.181 | 0.0181 | 1.83E-23 | 0.6173 | T | C | 757601 | 1.00E+02 | 1.32E-04 | DBP |
| rs13237249 | 0.1366 | 0.0177 | 1.03E-14 | 0.602 | T | C | 757601 | 5.96E+01 | 7.86E-05 | DBP |
| rs75511781 | -0.3721 | 0.047 | 2.45E-15 | 0.0425 | A | G | 757601 | 6.27E+01 | 8.27E-05 | DBP |
| rs7800558 | 0.096 | 0.0175 | 4.46E-08 | 0.4219 | T | C | 757601 | 3.01E+01 | 3.97E-05 | DBP |
| rs13240040 | 0.1186 | 0.019 | 3.98E-10 | 0.3164 | A | G | 757601 | 3.90E+01 | 5.14E-05 | DBP |
| rs3918226 | 0.6117 | 0.0329 | 5.31E-77 | 0.9187 | T | C | 757601 | 3.46E+02 | 4.56E-04 | DBP |
| rs4726006 | 0.1339 | 0.02 | 2.39E-11 | 0.7452 | A | G | 757601 | 4.48E+01 | 5.92E-05 | DBP |
| rs6464165 | -0.217 | 0.0195 | 7.34E-29 | 0.2809 | T | C | 757601 | 1.24E+02 | 1.63E-04 | DBP |
| rs9638084 | 0.1154 | 0.0178 | 8.51E-11 | 0.6022 | A | G | 757601 | 4.20E+01 | 5.55E-05 | DBP |
| rs17432462 | -0.1036 | 0.0179 | 7.31E-09 | 0.3766 | T | C | 757601 | 3.35E+01 | 4.42E-05 | DBP |
| rs6959688 | -0.1269 | 0.0178 | 1.02E-12 | 0.4015 | A | G | 757601 | 5.08E+01 | 6.71E-05 | DBP |
| rs2906152 | -0.1873 | 0.0181 | 5.55E-25 | 0.3696 | A | G | 757601 | 1.07E+02 | 1.41E-04 | DBP |
| rs4722548 | -0.1346 | 0.0176 | 1.99E-14 | 0.3996 | T | C | 757601 | 5.85E+01 | 7.72E-05 | DBP |
| rs3735533 | -0.487 | 0.0331 | 6.32E-49 | 0.9258 | T | C | 757601 | 2.16E+02 | 2.86E-04 | DBP |
| rs342977 | -0.1577 | 0.0205 | 1.67E-14 | 0.2285 | A | G | 757601 | 5.92E+01 | 7.81E-05 | DBP |
| rs17454517 | 0.1216 | 0.0174 | 2.65E-12 | 0.5064 | A | G | 757601 | 4.88E+01 | 6.45E-05 | DBP |
| rs1178979 | 0.1504 | 0.0221 | 9.96E-12 | 0.1953 | T | C | 757601 | 4.63E+01 | 6.11E-05 | DBP |
| rs5010183 | 0.1195 | 0.018 | 2.86E-11 | 0.372 | T | C | 757601 | 4.41E+01 | 5.82E-05 | DBP |
| rs3807101 | -0.1743 | 0.0265 | 4.57E-11 | 0.877 | T | C | 757601 | 4.33E+01 | 5.71E-05 | DBP |
| rs7788746 | -0.1644 | 0.0183 | 3.19E-19 | 0.3309 | T | G | 757601 | 8.07E+01 | 1.07E-04 | DBP |
| rs2978098 | 0.1548 | 0.0176 | 1.33E-18 | 0.4535 | A | C | 757601 | 7.74E+01 | 1.02E-04 | DBP |
| rs142449193 | -0.2573 | 0.0426 | 1.51E-09 | 0.954 | T | C | 757601 | 3.65E+01 | 4.82E-05 | DBP |
| rs2957468 | 0.1377 | 0.0185 | 8.43E-14 | 0.6646 | A | G | 757601 | 5.54E+01 | 7.31E-05 | DBP |
| rs35091929 | 0.1828 | 0.0177 | 6.46E-25 | 0.6032 | T | C | 757601 | 1.07E+02 | 1.41E-04 | DBP |
| rs722783 | -0.2093 | 0.0208 | 9.03E-24 | 0.7784 | A | G | 757601 | 1.01E+02 | 1.34E-04 | DBP |
| rs9918907 | -0.1188 | 0.021 | 1.59E-08 | 0.2162 | A | G | 757601 | 3.20E+01 | 4.22E-05 | DBP |
| rs7012891 | -0.1391 | 0.0205 | 1.20E-11 | 0.2367 | T | C | 757601 | 4.60E+01 | 6.08E-05 | DBP |
| rs4074812 | -0.1336 | 0.0175 | 2.07E-14 | 0.4465 | A | G | 757601 | 5.83E+01 | 7.69E-05 | DBP |
| rs3802230 | -0.1605 | 0.0174 | 2.75E-20 | 0.4554 | A | C | 757601 | 8.51E+01 | 1.12E-04 | DBP |
| rs62503324 | 0.2033 | 0.0204 | 2.11E-23 | 0.7603 | T | C | 757601 | 9.93E+01 | 1.31E-04 | DBP |
| rs17832905 | 0.1923 | 0.0346 | 2.81E-08 | 0.9283 | A | C | 757601 | 3.09E+01 | 4.08E-05 | DBP |
| rs17321041 | 0.2313 | 0.0363 | 1.78E-10 | 0.9367 | T | C | 757601 | 4.06E+01 | 5.36E-05 | DBP |
| rs1906672 | 0.1402 | 0.0205 | 8.48E-12 | 0.7676 | A | G | 757601 | 4.68E+01 | 6.17E-05 | DBP |
| rs10087280 | 0.1381 | 0.0232 | 2.54E-09 | 0.1683 | A | G | 757601 | 3.54E+01 | 4.68E-05 | DBP |
| rs4873492 | 0.1401 | 0.0231 | 1.28E-09 | 0.8275 | T | C | 757601 | 3.68E+01 | 4.86E-05 | DBP |
| rs2442618 | -0.1315 | 0.0177 | 1.21E-13 | 0.4277 | T | C | 757601 | 5.52E+01 | 7.29E-05 | DBP |
| rs11778153 | 0.1192 | 0.0182 | 5.84E-11 | 0.3569 | T | C | 757601 | 4.29E+01 | 5.66E-05 | DBP |
| rs6983239 | 0.1159 | 0.0211 | 3.71E-08 | 0.7812 | T | G | 757601 | 3.02E+01 | 3.98E-05 | DBP |
| rs148401029 | -0.3122 | 0.0486 | 1.32E-10 | 0.9648 | A | C | 757601 | 4.13E+01 | 5.45E-05 | DBP |
| rs56345595 | 0.1329 | 0.0177 | 5.20E-14 | 0.4152 | A | G | 757601 | 5.64E+01 | 7.44E-05 | DBP |
| rs4743021 | -0.108 | 0.0194 | 2.41E-08 | 0.3147 | T | C | 757601 | 3.10E+01 | 4.09E-05 | DBP |
| rs10980408 | -0.3745 | 0.0477 | 4.17E-15 | 0.0358 | T | C | 757601 | 6.16E+01 | 8.14E-05 | DBP |
| rs10759697 | 0.1308 | 0.0173 | 3.94E-14 | 0.5094 | A | G | 757601 | 5.72E+01 | 7.54E-05 | DBP |
| rs2133386 | -0.1322 | 0.0176 | 5.21E-14 | 0.5673 | A | C | 757601 | 5.64E+01 | 7.45E-05 | DBP |
| rs507666 | -0.2854 | 0.0223 | 2.27E-37 | 0.8128 | A | G | 757601 | 1.64E+02 | 2.16E-04 | DBP |
| rs6271 | -0.4313 | 0.0352 | 1.72E-34 | 0.9263 | T | C | 757601 | 1.50E+02 | 1.98E-04 | DBP |
| rs11145807 | 0.155 | 0.0184 | 4.10E-17 | 0.5942 | A | G | 757601 | 7.10E+01 | 9.37E-05 | DBP |
| rs4615669 | -0.114 | 0.0174 | 6.10E-11 | 0.4403 | A | G | 757601 | 4.29E+01 | 5.67E-05 | DBP |
| rs12216886 | 0.1292 | 0.0221 | 4.76E-09 | 0.1923 | T | G | 757601 | 3.42E+01 | 4.51E-05 | DBP |
| rs1243876 | -0.1063 | 0.019 | 2.14E-08 | 0.2988 | T | C | 757601 | 3.13E+01 | 4.13E-05 | DBP |
| rs76452347 | -0.2246 | 0.0229 | 9.37E-23 | 0.7947 | T | C | 757601 | 9.62E+01 | 1.27E-04 | DBP |
| rs12337056 | 0.1364 | 0.0228 | 2.18E-09 | 0.8239 | T | C | 757601 | 3.58E+01 | 4.72E-05 | DBP |
| rs11141731 | -0.1258 | 0.0207 | 1.31E-09 | 0.772 | T | C | 757601 | 3.69E+01 | 4.87E-05 | DBP |
| rs3916033 | -0.1233 | 0.0185 | 2.42E-11 | 0.4435 | T | C | 757601 | 4.44E+01 | 5.86E-05 | DBP |
| rs440454 | -0.2602 | 0.0192 | 7.52E-42 | 0.684 | A | G | 757601 | 1.84E+02 | 2.42E-04 | DBP |
| rs62064603 | -0.1335 | 0.0229 | 5.52E-09 | 0.8144 | T | C | 757601 | 3.40E+01 | 4.49E-05 | DBP |
| rs73033340 | 0.5312 | 0.0525 | 5.06E-24 | 0.0362 | A | G | 757601 | 1.02E+02 | 1.35E-04 | DBP |
| rs79889784 | -0.3941 | 0.0717 | 3.86E-08 | 0.9824 | T | G | 757601 | 3.02E+01 | 3.99E-05 | DBP |
| rs9508495 | -0.1944 | 0.0204 | 1.34E-21 | 0.2431 | T | C | 757601 | 9.08E+01 | 1.20E-04 | DBP |
| rs10764319 | 0.2690 | 0.0329 | 2.54E-16 | 6.96E-01 | T | C | 757601 | 6.69E+01 | 8.82E-05 | CCB |
| rs34606998 | 0.2580 | 0.0357 | 4.34E-13 | 7.61E-01 | T | C | 757601 | 5.22E+01 | 6.89E-05 | CCB |
| rs11012811 | 0.3100 | 0.0326 | 2.31E-21 | 6.90E-01 | T | G | 757601 | 9.04E+01 | 1.19E-04 | CCB |
| rs1888693 | 0.3860 | 0.0317 | 4.69E-34 | 6.55E-01 | A | G | 757601 | 1.48E+02 | 1.96E-04 | CCB |
| rs17604757 | -0.5020 | 0.0606 | 1.12E-16 | 6.75E-02 | A | G | 757601 | 6.86E+01 | 9.06E-05 | CCB |
| rs12571593 | -0.4000 | 0.0521 | 1.71E-14 | 9.27E-02 | A | G | 757601 | 5.89E+01 | 7.78E-05 | CCB |
| rs17662793 | 0.2360 | 0.0338 | 2.65E-12 | 2.88E-01 | A | G | 757601 | 4.88E+01 | 6.43E-05 | CCB |
| rs2482100 | 0.3130 | 0.0417 | 5.65E-14 | 8.43E-01 | A | G | 757601 | 5.63E+01 | 7.44E-05 | CCB |
| rs61278674 | -0.3300 | 0.0540 | 1.03E-09 | 9.38E-02 | A | G | 757601 | 3.73E+01 | 4.93E-05 | CCB |
| rs4748444 | 0.1940 | 0.0327 | 3.13E-09 | 3.36E-01 | T | C | 757601 | 3.52E+01 | 4.65E-05 | CCB |
| rs1779209 | 0.2740 | 0.0336 | 4.23E-16 | 7.12E-01 | T | C | 757601 | 6.65E+01 | 8.78E-05 | CCB |
| rs1757213 | 0.3080 | 0.0507 | 1.15E-09 | 8.88E-01 | A | G | 757601 | 3.69E+01 | 4.87E-05 | CCB |
| rs10828399 | -0.1950 | 0.0302 | 1.10E-10 | 4.78E-01 | A | G | 757601 | 4.17E+01 | 5.50E-05 | CCB |
| rs17610275 | 0.3870 | 0.0613 | 2.87E-10 | 7.34E-02 | T | G | 757601 | 3.99E+01 | 5.26E-05 | CCB |
| rs10828542 | 0.1820 | 0.0311 | 5.18E-09 | 3.86E-01 | A | G | 757601 | 3.42E+01 | 4.52E-05 | CCB |
| rs10741039 | 0.1720 | 0.0301 | 1.22E-08 | 4.76E-01 | A | C | 757601 | 3.27E+01 | 4.31E-05 | CCB |
| rs11013938 | -0.3270 | 0.0350 | 1.17E-20 | 7.45E-01 | C | G | 757601 | 8.73E+01 | 1.15E-04 | CCB |
| rs35241357 | -0.3080 | 0.0317 | 2.64E-22 | 3.52E-01 | A | G | 757601 | 9.44E+01 | 1.25E-04 | CCB |
| rs112133583 | -0.5550 | 0.0973 | 1.18E-08 | 9.70E-01 | T | C | 757601 | 3.25E+01 | 4.29E-05 | CCB |
| rs10828662 | -0.2880 | 0.0304 | 2.54E-21 | 4.41E-01 | T | C | 757601 | 8.98E+01 | 1.18E-04 | CCB |
| rs982003 | -0.2410 | 0.0351 | 6.21E-12 | 2.43E-01 | T | C | 757601 | 4.71E+01 | 6.22E-05 | CCB |
| rs1325990 | -0.3870 | 0.0302 | 1.09E-37 | 5.30E-01 | A | G | 757601 | 1.64E+02 | 2.17E-04 | CCB |
| rs11014170 | -0.6700 | 0.1150 | 5.61E-09 | 9.79E-01 | A | G | 757601 | 3.39E+01 | 4.48E-05 | CCB |
| rs67214975 | -0.4140 | 0.0307 | 1.42E-41 | 5.44E-01 | A | C | 757601 | 1.82E+02 | 2.40E-04 | CCB |
| rs7923191 | -0.3690 | 0.0376 | 1.10E-22 | 2.08E-01 | A | G | 757601 | 9.63E+01 | 1.27E-04 | CCB |
| rs12258967 | 0.6330 | 0.0337 | 1.08E-78 | 2.95E-01 | C | G | 757601 | 3.53E+02 | 4.65E-04 | CCB |
| rs72786098 | -0.5030 | 0.0883 | 1.18E-08 | 9.68E-01 | A | G | 757601 | 3.24E+01 | 4.28E-05 | CCB |
| rs116936375 | -0.5740 | 0.0810 | 1.40E-12 | 9.60E-01 | A | G | 757601 | 5.02E+01 | 6.63E-05 | CCB |
| rs1998822 | -0.1960 | 0.0343 | 1.15E-08 | 2.77E-01 | A | G | 757601 | 3.27E+01 | 4.31E-05 | CCB |
| rs10828749 | -0.3660 | 0.0309 | 2.27E-32 | 5.88E-01 | A | G | 757601 | 1.40E+02 | 1.85E-04 | CCB |
| rs7076247 | 0.2560 | 0.0309 | 1.33E-16 | 6.11E-01 | T | C | 757601 | 6.86E+01 | 9.06E-05 | CCB |
| rs4748472 | 0.3160 | 0.0319 | 4.04E-23 | 3.44E-01 | T | C | 757601 | 9.81E+01 | 1.30E-04 | CCB |
| rs12416030 | -0.2090 | 0.0381 | 4.32E-08 | 2.03E-01 | T | C | 757601 | 3.01E+01 | 3.97E-05 | CCB |
| rs12416052 | 0.1990 | 0.0311 | 1.59E-10 | 4.05E-01 | T | C | 757601 | 4.09E+01 | 5.40E-05 | CCB |
| rs4748476 | 0.2170 | 0.0365 | 2.89E-09 | 2.23E-01 | T | C | 757601 | 3.53E+01 | 4.67E-05 | CCB |
| rs2239046 | 0.2080 | 0.0322 | 9.58E-11 | 3.18E-01 | A | G | 757601 | 4.17E+01 | 5.51E-05 | CCB |
| rs714277 | 0.1990 | 0.0333 | 2.38E-09 | 7.17E-01 | T | C | 757601 | 3.57E+01 | 4.71E-05 | CCB |
| rs718448 | 0.2060 | 0.0346 | 2.43E-09 | 7.43E-01 | T | C | 757601 | 3.54E+01 | 4.68E-05 | CCB |
| rs312487 | 0.2190 | 0.0307 | 9.65E-13 | 5.22E-01 | T | C | 757601 | 5.09E+01 | 6.72E-05 | CCB |
| rs3821843 | 0.3370 | 0.0335 | 6.56E-24 | 3.19E-01 | A | G | 757601 | 1.01E+02 | 1.34E-04 | CCB |
| rs9311502 | -0.2460 | 0.0355 | 3.87E-12 | 2.39E-01 | T | C | 757601 | 4.80E+01 | 6.34E-05 | CCB |
| rs1547950 | -0.2150 | 0.0307 | 2.33E-12 | 4.62E-01 | T | C | 757601 | 4.90E+01 | 6.47E-05 | CCB |
| rs11709630 | 0.1930 | 0.0320 | 1.61E-09 | 3.63E-01 | T | G | 757601 | 3.64E+01 | 4.80E-05 | CCB |
| rs114987861 | 0.5290 | 0.0958 | 3.36E-08 | 9.72E-01 | A | G | 757601 | 3.05E+01 | 4.02E-05 | CCB |
| rs113210396 | -0.4340 | 0.0770 | 1.76E-08 | 9.55E-01 | T | G | 757601 | 3.18E+01 | 4.19E-05 | CCB |
| rs7340705 | -0.2430 | 0.0322 | 4.87E-14 | 3.27E-01 | T | C | 757601 | 5.70E+01 | 7.52E-05 | CCB |
| rs2633731 | -0.1960 | 0.0309 | 2.21E-10 | 6.04E-01 | T | C | 757601 | 4.02E+01 | 5.31E-05 | CCB |
| rs10463311 | -0.0854 | 0.0156 | 4.00E-08 | 0.7441 | T | C | 80610 | 3.00E+01 | 3.72E-04 | ALS |
| rs12973192 | 0.1205 | 0.0153 | 3.92E-15 | 0.3247 | G | C | 80610 | 6.20E+01 | 7.69E-04 | ALS |
| rs3849943 | -0.1764 | 0.0155 | 3.77E-30 | 0.7518 | T | C | 80610 | 1.30E+02 | 1.60E-03 | ALS |
| rs74654358 | 0.1976 | 0.0337 | 4.66E-09 | 0.0473 | A | G | 80610 | 3.44E+01 | 4.26E-04 | ALS |
| rs75087725 | 0.5145 | 0.0672 | 1.85E-14 | 0.0153 | A | C | 80610 | 5.86E+01 | 7.27E-04 | ALS |
